# Development and biological characterization of a clinical gene transfer vector for the treatment of MAK-associated retinitis pigmentosa

**DOI:** 10.1038/s41434-021-00291-5

**Published:** 2021-09-14

**Authors:** Budd A. Tucker, Erin R. Burnight, Cathryn M. Cranston, Mallory J. Ulferts, Meagan A. Luse, Trudi Westfall, C. Anthony Scott, Autumn Marsden, Katherine Gibson-Corley, Luke A. Wiley, Ian C. Han, Diane C. Slusarski, Robert F. Mullins, Edwin M. Stone

**Affiliations:** 1 Institute for Vision Research, Carver College of Medicine, University of Iowa, Iowa City, IA USA; 2 Department of Ophthalmology and Visual Sciences, Carver College of Medicine, University of Iowa, Iowa City, IA USA; 3 Department of Biology, College of Liberal Arts and Sciences, University of Iowa, Iowa City, IA USA; 4 Department of Microbiology and Immunology, Vanderbilt University Medical Center, Nashville, TN USA

**Keywords:** Neurological disorders, Cell biology, Gene expression

## Abstract

By combining next generation whole exome sequencing and induced pluripotent stem cell (iPSC) technology we found that an Alu repeat inserted in exon 9 of the MAK gene results in a loss of normal MAK transcript and development of human autosomal recessive retinitis pigmentosa (RP). Although a relatively rare cause of disease in the general population, the MAK variant is enriched in individuals of Jewish ancestry. In this population, 1 in 55 individuals are carriers and one third of all cases of recessive RP is caused by this gene. The purpose of this study was to determine if a viral gene augmentation strategy could be used to safely restore functional MAK protein as a step toward a treatment for early stage MAK-associated RP. Patient iPSC-derived photoreceptor precursor cells were generated and transduced with viral vectors containing the MAK transcript. One week after transduction, transcript and protein could be detected via rt-PCR and western blotting respectively. Using patient-derived fibroblast cells and mak knockdown zebra fish we demonstrate that over-expression of the retinal MAK transgene restored the cells ability to regulate primary cilia length. In addition, the visual defect in mak knockdown zebrafish was mitigated via treatment with the retinal MAK transgene. There was no evidence of local or systemic toxicity at 1-month or 3-months following subretinal delivery of clinical grade vector into wild type rats. The findings reported here will help pave the way for initiation of a phase 1 clinical trial for the treatment of patients with MAK-associated RP.

## Introduction

Male germ cell-associated kinase (MAK) is a protein that regulates the length of the primary cilium of a variety of different cell types [1]. Interestingly, despite its original description as a gene that is important in spermatogenesis, deletion of the *Mak* gene in mice does not affect fertility [2]. Instead, *Mak*-deficient animals develop a slowly progressive form of retinal degeneration characterized by elongation of photoreceptor cell connecting cilia and an abnormal electroretinogram [3].

In 2010 we discovered that mutations in the *MAK* gene were responsible for approximately one third of autosomal recessive retinitis pigmentosa (RP) occurring in individuals of Jewish ancestry [1]. Clinically, *MAK*-associated RP is characterized by adult-onset loss of the light-sensing photoreceptor cells of the outer neural retina. The disease often begins in the inferonasal aspect of the retina resulting in a characteristic loss of visual field superotemporally [4]. The human retina expresses two different transcripts of the *MAK* gene. The most abundant is retina-specific and contains a 75 bp exon 12 that is absent from the canonical transcript that is expressed in a variety of cell types throughout the body. The most common disease-causing *MAK* genotype in RP patients is a homozygous 353 bp insertion of an *Alu* repeat in exon 9, which results in a translational frameshift and premature termination of translation [1]. As both the canonical and retina-specific isoforms of the *MAK* gene contain exon 9, MAK protein is completely absent in the cells of patients with this genotype.

Recessive diseases that are characterized by a loss of gene function are particularly amenable to viral-mediated gene replacement; delivery of full-length *MAK* to photoreceptor cells before they succumb to the disease could potentially slow or prevent vision loss. The fact that *MAK*-associated RP is an adult onset disease that is highly enriched in individuals of Jewish ancestry makes *MAK-*associated RP an excellent candidate for gene therapy [4].

Recent FDA approval of the adeno-associated virus (AAV)-based gene therapeutic voretigene neparvovec for the treatment of *RPE65*-associated Leber congenital amaurosis has invigorated the retinal gene therapy community. There are currently gene therapy clinical trials underway for the treatment of several other inherited retinal disorders. Unfortunately, there are over 100 different genes whose dysfunction cause inherited retinal disease, and more than 40 genes that cause RP [4]. So, to make significant headway against these diseases as a group, rapid simultaneous development of low-cost gene-based therapeutics will be required.

One of the most time-consuming obstacles to the development of a retinal gene therapy is the identification of a model system that recapitulates the disease phenotype faithfully enough that it can be used to demonstrate treatment efficacy. To streamline this step, we have devised an AAV testing pipeline that uses patient iPSC-derived retinal cells for this purpose. This strategy enables simultaneous testing of multiple isoforms of the gene of interest as well as several different promoter systems in human cells, without the need for a large number of animals. Once the optimal genetic isoform and promoter combination is determined the construct can be packaged into the appropriate AAV serotype under cGMP conditions and injected into wildtype animals (i.e., Sprague Dawley rats) to evaluate safety at human relevant clinical doses.

In this study, we generated constructs driving either the canonical or the retinal-specific *MAK* using two different promoters: a stronger Cytomegalovirus (CMV) promoter and a weaker EF1α promoter. Each of these constructs was tested for their ability to drive expression of their corresponding *MAK* transcript and corresponding protein in patient-iPSC-derived photoreceptor precursor cells. Further, each construct was assessed for its ability to regulate primary cilia length in both patient-derived fibroblast cells in vitro and *mak* knockdown zebrafish in vivo. Following identification of the optimal *MAK* isoform and promoter combination, clinical grade virus was packaged under current Good Manufacturing Practice (cGMP) conditions and subjected to safety analysis in wildtype Sprague Dawley rats. Animals were sacrificed 1-month and 3-months following subretinal injection and subjected to full necropsy, histopathological investigation and hematology and clinical chemistry analysis (for hematology and clinical chemistry an additional 3-days post-injection group was included). Collectively, the findings reported in this manuscript will be useful for initiation of a phase 1 clinical trial for the treatment of patients with *MAK*-associated RP.

## Materials and methods

### Ethics statement

All patients provided written, informed consent for this study, which was approved by the Institutional Review Board of the University of Iowa (project approval # 200202022) and adhered to the tenets set forth in the Declaration of Helsinki. The three patients in this study were molecularly confirmed to be homozygous for the 353 bp Alu insertion in the *MAK* gene, which we have previously demonstrated to cause *MAK*-associated retinitis pigmentosa [1, 5]. All zebrafish and rat experiments were conducted with the approval of the University of Iowa Animal Care and Use Committee (Animal welfare assurance #8071513 and #1031317, respectively) and were consistent with the ARVO Statement for the Use of Animals in Ophthalmic and Vision Research and the Treaty of Helsinki.

### Patient-derived cells

Following informed consent, skin biopsies were collected from three patients with *MAK*-associated RP and used for fibroblast isolation and iPSC generation as described previously [6–8]. Briefly, dermal fibroblast cells were reprogrammed using the CytoTune non-integrating Sendai virus reprogramming kit according to the manufacturers protocol (Invitrogen/Thermo Fisher Scientific, Waltham, MA; CytoTune-iPS Reprogramming Kit; Cat#: A16517) [9]. Fibroblasts were plated on 6-well tissue culture plates and infected at a multiplicity of infection (MOI) of 5. At 12–16 h following transduction cells were washed and fed with fresh fibroblast cell growth media [(DMEM/F12, 5% heat inactivated FBS (Invitrogen/Thermo Fisher Scientific) and 0.2% primocin (Invivogen, San Diego, CA)]. At 7 days post-infection, cells were passaged onto 6-well LN521-coated cell culture dishes at a density of 30,000 cells/well and fed every day with E8 pluripotency media (Invitrogen/Thermo Fisher Scientific). At 3 weeks post-viral transduction, iPSC colonies were picked, passaged, and clonally expanded on fresh LN521-coated cell culture dishes. During reprogramming and maintenance of pluripotency, cells were cultured at 5% CO_2_, 10% O_2_, and 37 **°**C.

### Differentiation of iPSC-derived photoreceptor precursor cells

For ease of viral transduction, patient-derived iPSCs were differentiated under 2D conditions as described previously [6, 7]. Briefly iPSCs were harvested and cultured on ultra low-binding plates (Corning Life Sciences, Tewksbury, MA) in embryoid body formation medium [DMEM F-12 (Gibco/Thermo Fisher Scientific), 10% knockout serum replacement (Gibco/Thermo Fisher Scientific), 2% B27 supplement (Gibco/Thermo Fisher Scientific), 1% N2 supplement (Cell Therapy Systems/Thermo Fisher Scientific), 1% L-glutamine (Life Technologies/Thermo Fisher Scientific), 1X NEAA (Life Technologies/Thermo Fisher Scientific), 0.2% Primocin™ (Invivogen), 1 ng/ml DKK-1 (R&D Systems, Minneapolis, MN), 1 ng/ml IGF-1 (R&D Systems), 1 ng/ml Noggin (R&D Systems) and 0.5 ng/ml bFGF (R&D Systems)] for 4–5 days. Embryoid bodies (~50/well) were plated on 6-well plates (Corning Life Sciences) coated with 25 μg/ml collagen (BD Bioscience, San Jose, CA), 50 μg/ml laminin (Life Technologies/Thermo Fisher Scientific), and 100 μg/ml fibronectin (Sigma-Aldrich) and cultured in differentiation medium one [DMEM F-12 (Life Technologies/Thermo Fisher Scientific), 2% B27 supplement (Gibco/Thermo Fisher Scientific), 1% N2 supplement (Life Technologies/Thermo Fisher Scientific), 1% L-Glutamine (Life Technologies/Thermo Fisher Scientific), 1X NEAA (Life Technologies/Thermo Fisher Scientific), 0.2% Primocin™ (Invivogen), 10 ng/ml DKK-1 (R&D Systems), 10 ng/ml IGF-1 (R&D Systems), 10 ng/ml Noggin (R&D Systems) and 5 ng/ml bFGF (R&D Systems)]. Embryoid bodies were differentiated for 10 days in differentiation medium one, six days in differentiation medium two [(differentiation media one plus 10 μM DAPT (EMD Millipore, Billerica, MA)] and 12 days in differentiation medium three [(differentiation media two plus 2 ng/ml aFGF (R&D Systems)]. To generate photoreceptor precursor cells, cultures were fed every other day for an additional 60 days in differentiation medium four [DMEM F-12 (Life Technologies/Thermo Fisher Scientific), 2% B27 supplement (Life Technologies/Thermo Fisher Scientific), 1% N2 supplement (Life Technologies/Thermo Fisher Scientific), 1% L-Glutamine (Life Technologies/Thermo Fisher Scientific), 1X NEAA (Life Technologies/Thermo Fisher Scientific), 0.2% Primocin™ (Invivogen)]. Patient-specific cells were differentiated for a total of 90 days.

### Lentiviral transgene cassette cloning and packaging

For the fibroblast primary cilia rescue experiments, *MAK* constructs were delivered via lentiviral transduction because the efficiency of AAV-mediated transduction of human dermal fibroblast is usually quite low. Lentiviral vectors were generated as we described previously [10]. Briefly, DNA containing the coding sequence of either the human canonical *MAK* isoform (GenBank Accession No. NM_005906) or the human retinal-specific *MAK* isoform (GenBank Accession No. NM_001242957) were cloned into the Gateway^®^ gene entry vector pENTR3C (pENTR_*MAK*^*CI*^ or pENTR_*MAK*^*RI*^; Life Technologies/Thermo Fisher Scientific). HIV-1 transgene cassette plasmids expressing either the CMV- or the EF1α-promoter driving *MAK*^*CI*^ or *MAK*^*RI*^ were derived using three-plasmid LR recombination reactions including the Gateway^®^ promoter entry vectors pENTR5’/CMVp or pENTR5’/EF1α (Life Technologies/Thermo Fisher Scientific), the gene entry clones pENTR_*MAK*^*CI*^ or pENTR_*MAK*^*RI*^, and the Gateway^®^ destination clone pLenti6.4/R4R2/V5-DEST (Life Technologies/Thermo Fisher Scientific) according to the manufacturer’s instructions. Third generation lentiviral vectors were produced at the University of Iowa Gene Transfer Vector Core using the Invitrogen Corporation ViraPower™ Lentivirus Expression System (Invitrogen/Thermo Fisher Scientific)). HIV-1 packaging, transgene cassette, VSV-G envelope, and Rev protein expression plasmids were co-transfected into HEK293FT cells using Lipofectamine 2000 as previously described [10]. Collected supernatants were concentrated 250:1 via overnight centrifugation and reconstituted in alpha-lactose buffer (40 mg/ml in 1X PBS). Concentrated vector was titered on HT1080s and HIV-1 genomes were quantified using TaqMan® qPCR.

### Adeno-associated virus transgene cassette cloning and viral vector packaging

To generate AAV transgene cassette plasmids expressing either the canonical or retinal *MAK* isoform under control of either the CMV or EF1α promoter, cDNAs were cloned into the KpnI/NheI linearized pFBAAVmcsBgHpA AAV shuttle plasmid (University of Iowa Gene Transfer Vector Core). The resulting 4 plasmids carried either CMV-driven canonical *MAK* isoform (CMV- *MAK*^*CI*^), CMV-driven retina-specific *MAK* isoform (CMV- *MAK*^*RI*^), EF1α-driven canonical *MAK* isoform (EF1α-*MAK*^*CI*^)or EF1α-driven retina-specific *MAK* isoform (EF1α-*MAK*^*RI*^), which were flanked by AAV2 inverted terminal repeats. Recombinant AAV2/5 vectors were produced in the University of Iowa Gene Transfer Vector Core. Vectors were produced via triple transfection as described previously [11]. Physical titers were determined via TaqMan® qPCR. Vector purity was determined via silver staining [12].

### cGMP production of clinical grade AAV5-CMV-MAK^RI^ vectors

Production of clinical grade AAV vector was performed as we have previously described [11]. Briefly, AAV5-CMV-*MAK*^*RI*^ was manufactured under cGMP in the Steven W. Dezii Translational Vision Research Facility (DTVRF) within the Institute for Vision Research at the University of Iowa. This facility contains two independent suites: one dedicated to autologous iPSC-derived retinal cell generation and one dedicated to AAV production. Both suites contain high-efficiency HEPA-filtered ISO class 7 (class 10,000) gowning areas and ISO class 6 (class 1000) processing rooms. The AAV processing room is equipped with 2 six-foot biosafety cabinets monitored by real time particle counters that exceed ISO class 5 (class 100) cleanliness standards, 2 copper lined heracell incubators, an iCellis bioreactor and a dedicated support room separated by a sliding glass door that contains both benchtop and ultra-centrifuges. AAV5-CMV-*MAK*^*RI*^ was manufactured using a characterized human HEK293T master cell line and a triple transfection method. Specifically, HEK293T cells were transfected with a set of constructs encoding (1) normal human retina-specific *MAK*^*RI*^ driven by the CMV promoter (pAAV2-CMV- *MAK*^*RI*^, which contains the expression cassette flanked by AAV2 ITRs) and (2) AAV (pXX2-R5C5, AAV5 packaging plasmid containing AAV5 *rep* and *cap* sequences) and helper virus-derived sequences (pHelper, containing *E2A* and *E4* genes from adenovirus). Each of these plasmids were sequence confirmed via bidirectional sequencing before packaging in the DTVRF. HEK293T cells taken from a qualified Master Cell Bank (DTVRF HEK293T) were plated and expanded in T600 multilayer tissue culture flasks (production batch scale of 20 flasks). Upon reaching confluence, cells were simultaneously transfected with pAAV2-CMV-*MAK*^*RI*^, pXX2-R5C5, and pHelper. To purify AAV5-CMV-*MAK*^*RI*^, the following steps were performed: (1) HEK293T cultures were passaged and centrifuged to remove cell culture reagents and low molecular weight impurities; (2) HEK293T cells were lysed to release the intracellular vector, and nuclease digestion was performed to remove nucleic acid impurities; (3) cell debris was removed from the lysate via filtration, using 0.45- and 0.2-μm filters; (4) density gradient ultracentrifugation was performed to separate empty capsids (a major impurity) from the vector product; (5) affinity chromatography was performed to purify vector; (6) viral particles were concentrated via buffer exchange; and (7) working concentrations of AAV5-CMV-*MAK*^*RI*^ were formulated in storage and injection buffer (180 m*M* NaCl plus 10 m*M* Na_3_PO_4_ [trisodium phosphate] in water for injection at pH 7.2), filtered through a 0.2-μm filter, vialed and labeled.

### Viral transduction of cells

JK1 murine testicular stromal cells and patient iPSC-derived photoreceptor precursor cells were infected at a MOI of 10^4^ vector genomes (vg) per cell. Patient dermal fibroblast cells were transduced at a MOI of 5. Viral transduction was performed in serum free cell culture media. Specifically, for JK1 and patient fibroblast cells, FBS was removed from the culture media and for iPSC-derived photoreceptor precursor cells transduction was performed in complete differentiation media (as described above). At 16 h post-infection, cells were washed with fresh complete medium and fed every other day for 7 days. At 7 days post-infection, cells were harvested for analysis as appropriate. For ciliogenesis assays, cells were infected as above, allowed to recover for 3 days and then serum-starved for 72 h to induce primary cilia formation prior to analysis.

### Ciliogenesis assay in patient-derived dermal fibroblast cells

Dermal fibroblasts from three patients with *MAK*-associated RP (both with and without viral transduction) and an unaffected control individual were cultured on collagen-coated chamber slides in serum-free conditions [MEMα (Life Technologies/Thermo Fisher Scientific), 2% v/v Primocin (Life Technologies/Thermo Fisher Scientific)] for 72 h. Cells were fixed in 4% paraformaldehyde and methanol and stained with an antibody targeted against acetylated tubulin (1:200, Sigma-Aldrich). Slides were coverslipped with Polyvinyl alcohol (PVA) mounting medium containing 1,4-Diazabicyclo[2.2.2]octane (DABCO) (100 μg/ml PVA, Sigma-Aldrich; 25% v/v glycerol, Sigma-Aldrich; 0.1 M Tris-HCl, pH 8-8.5, 25 μg/ml DABCO, Sigma-Aldrich) and 4’,6-Diamidino-2-phenylindole dihydrochloride (DAPI) (Sigma-Aldrich; 1:10,000). Following antibody labeling, slides were coded to eliminate experimenter bias. Cells and cilia were imaged and counted by two individuals who were masked to the identity of the treatment groups. Cilia were imaged using a Leica DM 2500 SPE confocal microscope (Leica Microsystems, Wetzlar, Germany). Fields of cells were found using DAPI followed by imaging acetylated tubulin. Primary cilia were counted as elongated or punctate acetylated tubulin-positive structures localized to nuclei or immediately perinuclear.

### Quantification of cilia length

Cilia were measured using the Neurite tracer plugin in ImageJ as we described previously (24807808). Briefly, images of cells with primary cilia (*n* = 25 cells for each of three patients and a control individual) were collected via confocal microscopy using a 63× objective. Cilia displaying clear labeling of acetylated tubulin (axoneme) were traced and measurements were obtained.

### Immunoblotting for MAK

Western blots were performed as described previously [1, 5, 10]. Briefly, cells were treated with 0.25% Trypsin-EDTA (Life Technologies/Thermo Fisher Scientific), homogenized in lysis buffer [50 mM Tris-HCl, pH 7.6, 150 mM NaCl, 10 mM CaCl_2_, 1% triton X-100, 0.02% NaN_3_, (Sigma Aldrich)] and centrifuged. Supernatant protein concentrations were determined using bicinchoninic acid (BCA) according to manufacturer’s instructions (Pierce, Rockford, IL). Fifty micrograms each were subjected to SDS-PAGE (4-20% acrylamide), transferred to PVDF, and probed with rabbit anti-MAK antibody (Abcam, Cambridge, England; Cat. #: ab80536). Blots were visualized with ECL reagents (GE Healthcare Life Sciences, Pittsburgh, PA) and exposed to X-ray film (Fisher Scientific, Pittsburgh, PA).

### rt-PCR

RNA was isolated from cells using a RNeasy Mini Kit (Qiagen, Venlo, Limburg, the Netherlands) and the final concentration determined using a NanoDrop spectrophotometer (Thermo Fisher Scientific). cDNA was produced using a High Capacity cDNA Reverse Transcriptase Kit (Life Technologies/Thermo Fisher Scientific). Specific genes were then amplified using rt-PCR. The resulting DNA was characterized by electrophoresis on a 2% agarose gel for 30 min. Products were subsequently gel purified and sequence confirmed.

### Subretinal injection of AAV5-CMV-MAK^RI^

All animal procedures were approved by the University of Iowa’s Animal Care and Use Committee. 2-month-old wildtype Sprague Dawley rats (Charles River; *N* = 60, 30 M and 30 F) were anesthetized via isofluorane inhalation and treated with subretinal injection, as previously described [13, 14]. Half of the animals received a single 10 μl injection of purified and concentrated clinical-grade AAV5-CMV-*MAK*^*RI*^ (10^11^ vg) and half of the animals received an equal volume of AAV storage and injection buffer. Rats were sacrificed 1-month and 3-months post-injection. Hematology and clinical chemistry (*N* = 60, 30 M, and 30 F, performed by IDEXX, Columbia, MO) were performed at 3-day, 1-month, and 3-months post-injection. Rats sacrificed at 1-month and 3-months post-injection were evaluated with complete necropsy and histopathologic analysis following sectioning and H&E staining (*N* = 40, 20 M, and 20 F) were performed. All animals were examined at each time point by an ophthalmologist (ICH) to ensure no visible intraocular inflammation or other injection-related complications.

### Zebrafish mak expression domains

Zebrafish *mak* was isolated by PCR, cloned into the pCRII-TOPO TA vector (forward primer: 5′–cgctacgacctctgtttcct-3′, reverse primer: 5′-tgctctccttttccatccat-3′), and used to generate a riboprobe for whole mount in situ hybridization. The anti-sense probe was created by linearizing the plasmid with KpnI and transcribing the gene with T7 RNA polymerase. The sense probe was created by linearizing the plasmid with NotI and transcribing the gene with SP6 RNA polymerase. The Maxiscript RNA kit (Ambion), and DIG RNA labelling mix (Roche) were used for both probes. For retina sections, embryos were post-fixed after whole mount in situ hybridization in 4% paraformaldehyde followed by incubation for 1 h each at 4 °C in 15% sucrose in sterile H_2_O and 30% sucrose in sterile H_2_O. This was followed by overnight incubation at 4 °C in optimal cutting temperature compound (OCT). Embryos were oriented and frozen in OCT for cryosectioning at 12 µm and photographed using a Zeiss Axiophot compound microscope and Axiovision software at 20×, and 63×.

### Exogenous expression of Human MAK in zebrafish

Human myc-tagged *MAK* cDNAs were cloned into the pCS2 + expression vector. RNA generated by in vitro transcription was injected into live 1–2 cell stage zebrafish embryos. Protein lysates were isolated at selected developmental time points for Western blotting and probed with mouse anti-myc (9E10; Santa Cruz Biotechnology, Santa Cruz, CA at 1:10 000) and mouse anti-β-actin (1:2000, Sigma Clone AC-74, St Louis, MO) antibodies as previously described [15].

### Zebrafish knockdown and rescue

Morpholinos (MO) were designed to target the initiator methionine codon (AUG) and two different splice sites (*mak*_AUG: CTTGAGTGTCGTGTAACGGTTCATT; *mak*_exon 3: TGTGTTCACTGAGTTTGCACCTTGA; *mak*_exon 4: TGATATAGAATCTCATCATACCTGT). Control MO or *mak* MOs (2–3 ng) were injected into 1–2 cell stage zebrafish embryos. Sequential injection was used for rescue with 1-cell stage embryos being injected with MO and then separated into groups. One MO-injected group was injected with human *MAK* mRNA and the other group was not injected again and served as the MO-only control. Phenotypic analysis of these groups, along with RNA only and uninjected siblings, was performed by evaluators masked to the identity of the experimental conditions.

### Analysis of Kupffer’s Vesicle (KV) cilia length

Embryos at the 10-12 somite stage were fixed in 4% paraformaldehyde. Cilia were decorated with anti-acteylated tubulin (1:800, Sigma) followed with Alexa 563 Goat anti-mouse secondary (1:400, Molecular Probes). Tailbuds were mounted using Vectashield mounting medium and imaged using a Leica (SP2) confocal microscope (63X oil with 1 micron sections). Individual cilia in a MAX projection of a 10 µm optic section were traced using the Leica Confocal Software measurement tool. The lengths of all traced cilia were exported to excel for statistical analysis.

### Visual startle response

Zebrafish visual function was tested at 5 days-post-fertilization (dpf) using VIZN as previously described [16, 17]. Only larva with normal morphology and swim behaviors were used for the vision assay.

### Statistical analysis

One-way ANOVA with a Tukey’s post-hoc testing was used to determine statistical significance between treatment groups. P-values of less than 0.05 were considered statistically significant.

## Results

### Generation of AAV constructs that drive expression of canonical and retina-specific *MAK*

In 2010, we discovered that insertion of a 353 bp *Alu* repeat into exon 9 of the *MAK* gene is a significant cause of RP in patients of Jewish ancestry [1]. This mutation was found to cause a shift in the reading frame of the gene and creation of a premature stop codon resulting in loss of both canonical and retinal MAK expression in patient-derived photoreceptor precursor cells [1]. Loss of functional MAK protein, which normally acts as a negative regulator of cilia length, was found to cause elongation of the photoreceptor cell connecting cilia and progressive retinal degeneration in *Mak* knockout mice [3].

**Fig. 1:**
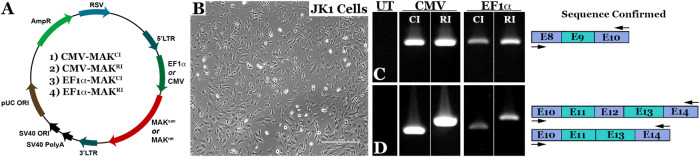
Design and generation of *MAK* gene transfer constructs. **A** Schematic depicting the four AAV constructs generated in this study. Constructs were designed to deliver either the human canonical (*MAK*^*CI*^ - lacks exon 12, constructs 1 and 3) or retina-specific *MAK* isoform (*MAK*^*RI*^ - contains exon 12, constructs 2 and 4) under control of either the constitutively active CMV or EF1α promoter. **B** Representative image of cultured murine JK1 cells (immortalized SMA + , CD34 + testicular stromal cells, which lack endogenous expression of human *MAK*). Scale bar 100 μm. **C**, **D** rt-PCR analysis performed on RNA isolated from JK1 cells transduced with either AAV5-CMV*-MAK*^*CI*^ (Construct 1), AAV5-CMV*-MAK*^*RI*^ (Construct 2), AAV5-EF1α*-MAK*^*CI*^ (Construct 3), or AAV5-EF1α*-MAK*^*RI*^ (Construct 4). Each construct is capable of driving robust expression of exon-9-containing human *MAK* transcript (**C**). Unlike the *MAK*^*CI*^ constructs, both AAV5-CMV*-MAK*^*RI*^ and AAV5-EF1α*-MAK*^*RI*^ constructs are capable of driving expression of the exon-12-containing retina-specific *MAK* transcript (**D**). Arrows denote rt-PCR primer positions.

The fact that human *MAK*-associated RP is a late onset recessive disorder and enriched in an identifiable population [4] makes it a good candidate for clinical gene augmentation. However, *MAK* has several isoforms, two of which, the canonical and retina-specific versions, are expressed in the retina. To determine whether AAV-mediated delivery of either canonical (lacking exon 12) or retinal-specific *MAK* (including exon 12) is capable of restoring functional *MAK* transcript and protein, we engineered AAV constructs in which expression of *MAK* is driven under control of two different constitutively active promoters: the stronger Cytomegalovirus (CMV) promoter and a weaker elongation factor 1 alpha (EF1α) promoter. The resulting four vectors are depicted in Fig. 1A: (1) AAV-CMV-*MAK*^*CI*^ (*MAK*^*CI*^ *-* canonical isoform, which includes exon 9 and lacks exon 12); (2) AAV-CMV-*MAK*^*RI*^ (*MAK*^*RI*^ - retina-specific isoform, which includes both exons 9 and 12); (3) AAV- EF1α-*MAK*^*CI*^; and (4) AAV- EF1α-*MAK*^*RI*^.

To evaluate the ability of each of these constructs to drive expression of canonical or retina-specific *MAK* transcript, we first packaged them into AAV5 particles via triple transfection of HEK293t cells and then used them to transduce mouse JK1 testicular stromal cells (Fig. 1B), which lack endogenous expression of the human *MAK* gene. We then used rt-PCR with primers spanning exon 9 to evaluate the ability of each vector to drive expression of the exon-9-containing *MAK* transcript (Fig. 1C). As expected, *MAK* expression was higher in cultures transduced with vectors containing the CMV promoter than those transduced with vectors containing the weaker EF1α promoter. Interestingly, regardless of promoter used, there appeared to be increased *MAK* expression in cultures transduced with vectors containing the retina-specific *MAK* isoform that includes the 75 bp retina-specific exon 12 than those transduced with the vectors containing the canonical *MAK* isoform that lack exon 12. A similar trend was seen when rt-PCR primers were placed in exons 10 and 14 (Fig. 1D). As expected, rt-PCR products generated following transduction with vectors containing the retina-specific *MAK* isoform were 75 bp larger than the products generated following transduction with vectors containing the canonical MAK isoform. Again, MAK expression was much more robust when driven under control of the CMV promoter than the EF1α promoter. Collectively, these findings demonstrate that we have successfully engineered AAV constructs capable of driving expression of both the human canonical and retina-specific *MAK* isoform.

### AAV5-mediated restoration of canonical and retinal-specific *MAK* transcript and protein in patient-specific iPSC-derived photoreceptor precursor cells

**Fig. 2:**
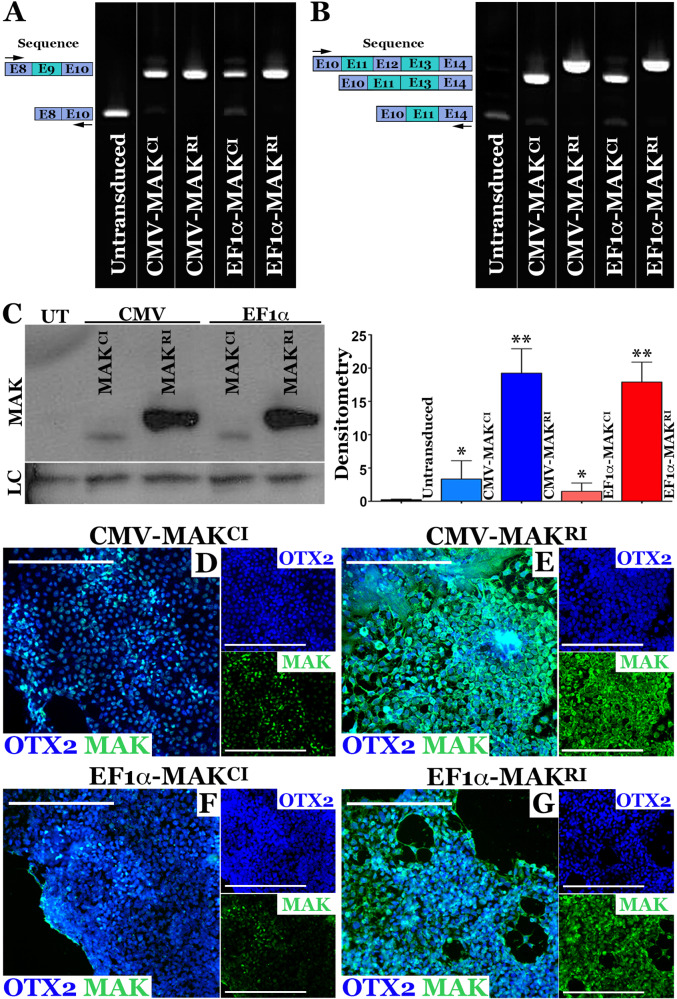
AAV5 mediated restoration of *MAK* transcript and protein in patient iPSC-derived photoreceptor precursor cells. **A**, **B** rt-PCR analysis performed on RNA isolated from patient-specific photoreceptor precursor cells two-weeks following transduction with AAV5-CMV*-MAK*^*CI*^, AAV5-CMV*-MAK*^*RI*^, AAV5-EF1α*-MAK*^*CI*^, or AAV5-EF1α*-MAK*^*RI*^ (MOI = 10^4^vg/cell). Compared to untransduced cultures (UT), which lack expression of wildtype exon-9-containing *MAK* transcript, cells transduced with each of the AAV5 constructs expressed exon-9-containing *MAK* transcripts. Only AAV5-CMV*-MAK*^*RI*^ and AAV5-EF1α*-MAK*^*RI*^ were capable of driving expression of exon-12-containing retinal *MAK* (**B**). **C** Western blot of patient-specific photoreceptor precursor cells transduced with AAV5-CMV*-MAK*^*CI*^, AAV5-CMV*-MAK*^*RI*^, AAV5-EF1α*-MAK*^*CI*^, or AAV5-EF1α*-MAK*^*RI*^ (MOI = 10^4^vg/cell). Only the constructs driving *MAK* exon 12 restored expression of full-length retinal-specific MAK protein. **D-G** Immunocytochemical analysis of patient iPSC-derived photoreceptor precursor cells following AAV5 transduction using antibodies targeted against MAK and OTX2 (photoreceptor precursor cell marker). Although MAK was detected in cultures transduced with AAV5 vectors carrying the canonical isoform (AAV5-CMV*-MAK*^*CI*^ (**D**) and AAV5-EF1α*-MAK*^*CI*^ (**F**)), pronounced expression throughout the cell body and neurites was only detected in cultures transduced with vectors carrying the retinal isoform (AAV5-CMV*-MAK*^*RI*^ (**E**) or AAV5-EF1α*-MAK*^*RI*^ (**G**)). MAK – green, OTX2 – blue. Scale bars = 200 μm. Arrows denote rt-PCR primer positions.

After demonstrating the ability of the AAV vectors described in Fig. 1 to drive *MAK* expression in a mouse cell line, we next sought to determine if we could restore *MAK* transcript and protein in a disease-relevant cell type, namely patient-iPSC-derived retinal photoreceptor precursor cells. As we have previously shown, iPSC-derived retinal cells generated from a patient molecularly confirmed to harbor homozygous *Alu* insertions in exon 9 of the *MAK* gene, lack expression of both exon 9- and exon 12-containing *MAK* transcripts and proteins [1]. For this experiment we differentiated patient-derived iPSCs for 90-days, the timepoint that we have previously shown photoreceptor precursor cells to be prominent [1, 7]. Cultures were subsequently transduced with each of the four *MAK* AAV constructs at a MOI of 10^4^ vg/cell. Rt-PCR analysis using primers spanning exon 9 (i.e., exons 8–10) of the *MAK* gene showed that compared to untransduced cells from the same patient that lack expression of an exon 9 containing transcript, each construct was capable of restoring exon 9 expression (Fig. 2A). Rt-PCR analysis using primers spanning exon 12 (i.e., exons 10–14) revealed that only constructs carrying the retina-specific *MAK* isoform (*MAK*^*RI*^) were able to restore expression of the exon 12 containing transcript, which was also absent in the untransduced cells from the same patient (Fig. 2B). Again, *MAK* transcripts driven under control of the CMV promoter were expressed at higher levels than those driven under control of the EF1α promoter (Fig. 2A, B). As anticipated, restoration of *MAK* transcript resulted in a restoration of MAK protein. Specifically, vectors containing the canonical *MAK* isoform successfully restored canonical MAK protein (Fig. 2C, *P < 0.05) and vectors containing the retinal *MAK* isoform successfully restored the retinal MAK protein (Fig. 2C, *P < 0.001). Although a difference in protein expression between CMV and EF1α containing constructs was visible, the difference was significantly less than the difference observed at the transcriptional level and did not reach statistical significance. However, the difference in protein expression detected following transduction with vectors driving expression of the retina-specific MAK isoform versus the canonical MAK isoform was significant (Fig. 2C, ***P < 0.001).

To confirm these Western blotting results and evaluate the pattern of cellular expression following viral transduction we next performed immunocytochemical analysis on patient-iPSC-derived photoreceptor precursor cell cultures. In this analysis, the human specific anti-MAK antibody used in Fig. 2C and an antibody targeted against the photoreceptor precursor cell transcription factor OTX2 were used (Fig. 2D–G). Again, the highest level of MAK expression was detected in cultures transduced with the retina-specific MAK isoform under control of the CMV promoter (Fig. 2E, AAV5-CMV-*MAK*^*RI*^*)*. This was followed by cultures transduced with vector containing the retinal *MAK* isoform under control of the EF1α promoter (Fig. 2G, AAV5-EF1α-*MAK*^*RI*^), the canonical MAK isoform under control of the CMV promoter (Fig. 2D, AAV5-CMV-*MAK*^*CI*^) and finally the canonical MAK isoform under control of the EF1α promoter (Fig. 2F, AAV5-EF1α-*MAK*^*CI*^). The pattern of MAK expression following transduction with each of the 4 vectors was strikingly different. Cells transduced with vectors containing the retina-specific isoforms had more robust levels of MAK expression throughout the cytoplasm extending into the neurites of OTX2-positive photoreceptor precursor cells (Fig. 2E, G). Canonical MAK protein appeared to be restricted to the nuclear/perinuclear region with minimal extension into neurites (Fig. 2D, F). Together, these results demonstrate that we were able to successfully restore full-length *MAK* transcript and protein in patient-iPSC-derived photoreceptor precursor cells using the *MAK* vectors we developed. Expression was more robust and widespread when the retina-specific MAK isoforms were delivered. Differences in protein expression levels between vectors containing the CMV and EF1α promoters were less pronounced than differences in protein expression levels observed between vectors carrying the canonical versus retinal *MAK* isoforms.

### Viral-mediated rescue of the primary cilia defect in patient-derived dermal fibroblasts

**Fig. 3:**
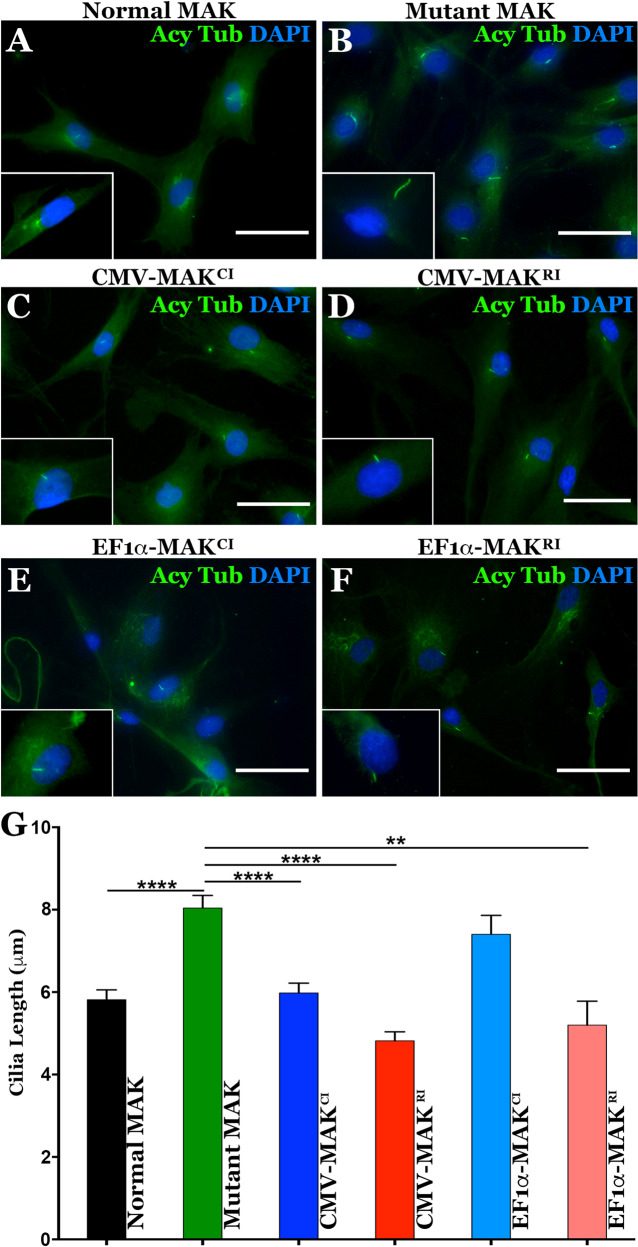
Restoration of MAK protein rescues aberrant primary cilia length defect in patient-derived dermal fibroblasts. **A–F** Immunocytochemical analysis using an antibody targeted against anti-acetylated tubulin (primary cilia marker) to stain dermal fibroblasts isolated from an unaffected control individual (A – control) and a patient with molecularly confirmed *MAK*-associated RP (**B**–**F**). Cells from the patient with *MAK*-associated RP were transduced with viral vectors driving canonical (**C**, **E**) or retinal *MAK* (D and **G**. Untransduced cultures of patient derived cells were used as a disease phenotype control (**B**). **G** Histogram comparing mean primary cilia length between a normal individual (*n* = 25 cells) and 3 independent patients with *MAK*-associated RP before and after transduction with viral vectors driving either canonical or retinal *MAK* (*n* = 75 cells, 25 cells per patient for each of 3 patients). The primary cilium in fibroblasts isolated from patients with *MAK*-associated RP was significantly longer than that of an unaffected control individual. Transduction with retinal *MAK* under control of either the CMV or EF1α promoter reduced the primary cilium length to that of the control. A significant reduction in primary cilium length was detected following transduction of with canonical *MAK* driven by the CMV promoter only. (**p < 0.01, ****P < 0.001). Scale bars = 100 μm.

Following confirmation that our *MAK* vectors were capable of driving expression of their respective *MAK* isoforms, we next asked if the resulting MAK proteins were functional. As indicated above, MAK functions as a negative regulator of primary cilia length. In mice, loss of MAK expression results in elongation of the connecting cilia in photoreceptor cells [3]. To determine if loss of the MAK protein in patients with *MAK*-associated RP had an effect on cilia length regulation, and if AAV5-mediated overexpression of MAK protein was sufficient to restore normal cilia length, an in vitro ciliogenesis assay was performed. We have previously demonstrated that serum starved patient-derived fibroblasts, whose primary cilia are readily detectible via immunocytochemical staining of acetylated tubulin, are an excellent model system for evaluating the effect of genetic defects on cilia formation and elongation [10]. In the present study, dermal fibroblasts were obtained from 3 independent patients with *MAK*-associated RP, each with homozygous *Alu* insertions in exon 9 of the *MAK* gene. Dermal fibroblasts isolated from a non-diseased individual were included as an unaffected control. Compared to the unffected individual, dermal fibroblasts isolated from patients with *MAK*-associated RP had significantly longer primary cilia (Fig. 3A vs. B, G, **P < 0.001). To determine if overexpression of canonical or retinal MAK protein could rescue the cilia length defect in patient-derived dermal fibroblasts, cultures were transduced with viral vectors driving expression of both canonical and retinal MAK under control of either the CMV or EF1α promoter as described above. As demonstrated in Fig. 3, the greatest effect on primary cilia length occurred following over-expression of the retina-specific isoform under control of the CMV promoter (Fig. 3D, G, *P* < 0.001), consistent with results described in Fig. 2 above. This was followed by the retina-specific isoform under control of the EF1α promoter (Fig. 3F, G, *P* < 0.01) and the canonical isoform under control of the CMV promoter (Fig. 3C, G, *P* < 0.001). Although a slight reduction in mean cilia length was detected in cells transduced with the canonical isoform driven under control of the EF1α promoter, this reduction did not reach statistical significance (Fig. 3E, G, *P* > 0.05). These experiments demonstrate that viral mediated delivery of retinal *MAK* under control of either the CMV or EF1α promoter is sufficient to restore a cell’s ability to properly regulate primary cilia length. Although canonical *MAK* did not significantly reduce primary cilia length when placed under control of the weaker EF1α promoter, the fact that it was capable of significantly reducing primary cilia length when placed under control of the stronger CMV promoter suggest that the function of canonical *MAK* in primary cilia is similar to that of the retinal-isoform. The difference in the two isoforms appear to be at the level of transcript expression and protein translation.

### Injection of *MAK* mRNA restores Kupffer’s vesicle cilia length and response to a visual stimulus in a zebrafish model of *MAK*-associated RP

**Fig. 4:**
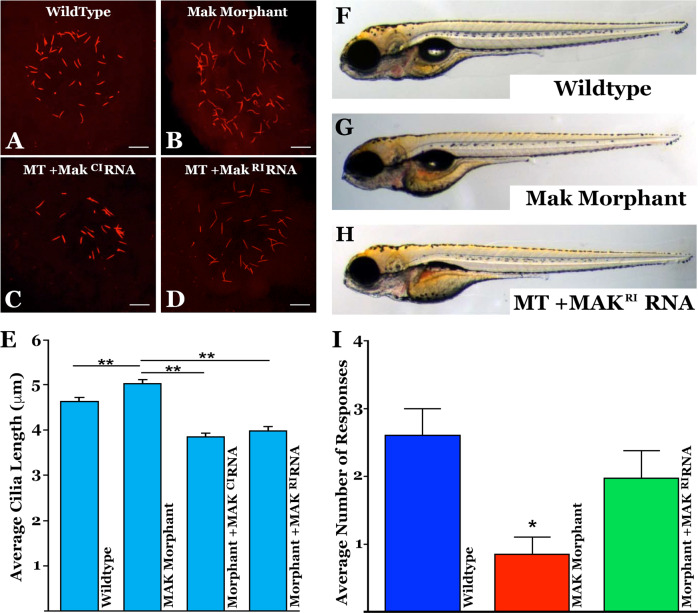
Injection of *MAK* mRNA restores Kupffer’s vesicle cilia length and response to visual stimulus in a *mak* mutant zebrafish model. **A**–**D** Immunocytochemical labeling of primary cilia with an anti-acetylated tubulin antibody within Kupffer’s vesicle in uninjected (**A**), *mak* morpholino-injected (**B**), and morpholino-injected zebrafish that simultaneously received canonical *MAK*^*CI*^ (**D**) or retinal *MAK*^*RI*^ mRNA (**D**). **E** Quantification of mean cilia length measured in each treatment group shown in **A**–**D**. *MAK* morphants displayed significantly longer cilia compared to uninjected siblings (uninjected vs MO only; *p* = 0.0004). Sequential injection of retinal *MAK*^*RI*^ mRNA or canonical *MAK*^*CI*^ mRNA significantly shortened primary cilia length (MO only vs MO + RNA; p < 0.0001 for both). **F**–**H** Light micrographs of wildtype (**F**), *MAK* morpholino-injected (**G**), and *MAK* morpholino and retinal *MAK* mRNA injected (**H**) demonstrating normal overall morphology among groups. **I** Histogram depicting the number of responses in the vision startle assay of wild-type, *MAK* morpholino-injected (MAK Mutant) and *MAK* morpholino/*MAK*^*RI*^ mRNA injected (Mutant + MAK^RI^ mRNA). *MAK* morphants had a significant decrease in average number of responses compared to wild-type fish (Wildtype vs MAK Mutant; *p* < 0.05). Injection of retinal *MAK* mRNA into *MAK* mutants partially rescued visual responses (i.e. no significant difference between wild-type and treatment groups). Kruskal-Wallis test with Dunn’s multiple comparisons. Scale bars in A-D = 10 μm.

To test whether delivery of canonical or retina-specific *MAK* mRNA could restore cilia length in vivo, a *mak*-knockdown zebrafish model was created. First, we evaluated the endogenous activity of zebrafish *mak*. Antisense morpholino oligonucleotides directed against the translation start site and splice junctions were used to knockdown zebrafish *mak* gene function (morphants). To measure cilia length, we assessed cilia within Kupffer’s vesicle (a small ciliated organ that is transiently present during early zebrafish embryogenesis). As in human patient-derived dermal fibroblasts, loss of *mak* resulted in significant elongation of the primary cilia (Fig. 4A vs B & E, *p* < 0.01). Three independent morpholinos directed against zebrafish *mak* all showed an increased cilia length defect.

To determine if overexpression of human *MAK* was capable of rescuing the Kupffer’s vesicle cilia length defect in mutant zebrafish, myc-tagged human *MAK* mRNA was generated. Sequential injection (at the single cell stage) of morphant zebrafish with human *MAK* mRNA demonstrated that the canonical and retina-specific *MAK* isoforms were capable of significantly reducing cilia length (Fig. 4B–D & E, *p* < 0.01). Consistent with the enriched *mak* expression domains in zebrafish, morphants displayed Kupffer’s vesicle cilia defects with no other gross morphological differences observed between wild-type (Fig. 4F), *mak* morpholino-injected (Fig. 4G), and *MAK* mRNA-injected fish (Fig. 4H). These results demonstrate that exogenous human *MAK* is sufficient to suppress the cilia length defect detected in *mak* mutant zebrafish without causing global morphological changes.

To determine if *mak* knockdown in zebrafish alters visual function, behavioral analysis of the vision-dependent startle response was performed. This assay is based on the observation that zebrafish display a specific swimming behavior when exposed to a rapid change in light that is lost in visually impaired fish [15, 18, 19]. As shown in Fig. 4I, when compared to wildtype fish, *mak* mutants display a significantly reduced startle response (*p* < 0.01). As described in the Kupffer’s vesicle experiment above, to evaluate rescue mRNA was injected into morphant zebrafish at the single cell stage. As injected mRNA is halved with each cell division, we expected that MAK protein would be diluted beyond the level of detection at the point we test for visual function at day 5. To our surprise, compared to *mak* morphants, zebrafish that were sequentially injected with retina-specific human *MAK* mRNA showed a significant increase in the average number of responses to visual stimuli, suggesting partial recovery of visual function (Fig. 4I, *p* < 0.05).

### Safety profile of clinical grade AAV5-CMV-*MAK*^*RI*^

Following analysis in patient-derived cells in vitro and *mak* morphant zebrafish in vivo, it became clear that of the four constructs we developed, AAV5-CMV-*MAK*^*RI*^ produced the most consistent and robust expression of functional MAK protein. This construct was therefore advanced through our clinical AAV production and preclinical local and systemic toxicity analysis pipeline. Following production under cGMP conditions, 10 μl of AAV5-CMV-*MAK*^*RI*^ viral particles (10^13^ vg/ml, 10^11^ vg per 10 μl injection) were injected into the subretinal space of 20 (10 M and 10 F) normal Sprague Dawley rats. As a control, an additional 20 (10 M and 10 F) animals received subretinal injections of an equal volume of AAV storage and injection buffer (vehicle). Half of the animals (i.e., 10 AAV5-CMV-*MAK*^*RI*^ treated (5 M and 5 F) and 10 vehicle control (5 M and 5 F)) were sacrificed 1-month following injection and the other half were sacrificed 3-months following injection. Following sacrifice, necropsy was performed, and gross findings recorded. The tissues listed in Supplemental Table 1 were collected, fixed, paraffin embedded, sectioned, stained with hematoxylin and eosin (H&E) and subjected to histopathological analysis by a certified veterinarian pathologist (please see Supplemental Fig. 1 for example H&E stained sections obtained from an animal at 3-months following subretinal injection of AAV5-CMV-*MAK*^*RI*^). As shown in Table 1, there were no significant AAV5-CMV-*MAK*^*RI*^ related findings noted at the time of necropsy. No significant AAV5-CMV-*MAK*^*RI*^ induced difference in mean organ weight or total body weight between treated and vehicle control animals at either of the timepoints tested were detected. Finally, no significant AAV5-CMV-*MAK*^*RI*^ related adverse findings were reported following complete histopathological analysis.

**Table 1:** Systemic Toxicity gross and histopathology analysis.

3-month male body weights						
Animal ID	Sex	Time point	Treatment group	Weight at surgery (g)	Weight at euthanasia (g)	Weight gain (g)
18R0184	M	3 M	rAAV Storage Buffer	85	545	460
18R0190	M	3 M	rAAV Storage Buffer	98	539	441
18R0191	M	3 M	rAAV Storage Buffer	98	616	518
18R0192	M	3 M	rAAV Storage Buffer	98	592	494
18R0193	M	3 M	rAAV Storage Buffer	105	642	537
Mean				96.80	586.80	490.00
Standard deviation				7.26	44.61	39.72
18R0180	M	3 M	DTVRF-AAV5-MAKret Lot#08292017	98	435	337
18R0181	M	3 M	DTVRF-AAV5-MAKret Lot#08292017	98	540	442
18R0182	M	3 M	DTVRF-AAV5-MAKret Lot#08292017	95	584	489
18R0183	M	3 M	DTVRF-AAV5-MAKret Lot#08292017	96	577	481
18R0194	M	3 M	DTVRF-AAV5-MAKret Lot#08292017	114	604	490
Mean				100.20	548.00	447.80
Standard deviation				7.82	67.28	64.98
*P*-value				0.8761	0.5411	0.4192
Significance				n.s.	n.s.	n.s.

3-month female body weights						
Animal ID	Sex	Time point	Treatment group	Weight at surgery (g)	Weight at euthanasia (g)	Weight gain (g)
18R0195	F	3 M	rAAV Storage Buffer	116	333	217
18R0196	F	3 M	rAAV Storage Buffer	114	320	206
18R0197	F	3 M	rAAV Storage Buffer	107	317	210
18R0198	F	3 M	rAAV Storage Buffer	102	289	187
18R0199	F	3 M	rAAV Storage Buffer	113	312	199
Mean				110.40	314.20	203.80
Standard deviation				5.77	16.08	11.43
18R0185	F	3 M	DTVRF-AAV5-MAKret Lot#08292017	92	267	175
18R0186	F	3 M	DTVRF-AAV5-MAKret Lot#08292017	94	345	251
18R0187	F	3 M	DTVRF-AAV5-MAKret Lot#08292017	103	320	217
18R0188	F	3 M	DTVRF-AAV5-MAKret Lot#08292017	103	366	263
18R0189	F	3 M	DTVRF-AAV5-MAKret Lot#08292017	111	318	207
Mean				100.60	323.20	222.60
Standard deviation				7.70	37.09	35.28
*P*-value				0.1778	0.9887	0.8949
Significance				n.s.	n.s.	n.s.

1-month male body weights						
Animal ID	Sex	Time point	Treatment group	Weight at surgery (g)	Weight at euthanasia (g)	Weight gain (g)
18R0091	M	1 M	rAAV Storage Buffer	180	399	219
18R0104	M	1 M	rAAV Storage Buffer	180	393	213
18R0105	M	1 M	rAAV Storage Buffer	166	447	281
18R0108	M	1 M	rAAV Storage Buffer	230	416	186
18R0109	M	1 M	rAAV Storage Buffer	240	452	212
Mean				199.20	421.40	222.20
Standard deviation				33.36	27.06	35.24
18R0092	M	1 M	DTVRF-AAV5-MAKret Lot#08292017	163	386	223
18R0095	M	1 M	DTVRF-AAV5-MAKret Lot#08292017	192	463	271
18R0096	M	1 M	DTVRF-AAV5-MAKret Lot#08292017	190	428	238
18R0100	M	1 M	DTVRF-AAV5-MAKret Lot#08292017	260	475	215
18R0101	M	1 M	DTVRF-AAV5-MAKret Lot#08292017	224	456	232
Mean				205.80	441.60	235.80
Standard deviation				37.22	35.56	21.53
*P*-value				0.9767	0.5530	0.7814
Significance				n.s.	n.s.	n.s.

1-month female body weights						
Animal ID	Sex	Time point	Treatment group	Weight at surgery (g)	Weight at euthanasia (g)	Weight gain (g)
18R0098	F	1 M	rAAV Storage Buffer	180	252	72
18R0102	F	1 M	rAAV Storage Buffer	160	256	96
18R0103	F	1 M	rAAV Storage Buffer	170	275	105
18R0106	F	1 M	rAAV Storage Buffer	155	254	99
18R0107	F	1 M	rAAV Storage Buffer	152	236	84
Mean				163.40	254.60	91.20
Standard deviation				11.52	13.89	13.18
18R0089	F	1 M	DTVRF-AAV5-MAKret Lot#08292017	162	255	93
18R0090	F	1 M	DTVRF-AAV5-MAKret Lot#08292017	162	258	96
18R0093	F	1 M	DTVRF-AAV5-MAKret Lot#08292017	172	235	63
18R0094	F	1 M	DTVRF-AAV5-MAKret Lot#08292017	170	243	73
18R0097	F	1 M	DTVRF-AAV5-MAKret Lot#08292017	167	245	78
Mean				166.60	247.20	80.60
Standard deviation				4.56	9.34	13.83
*P*-value				0.9972	0.9601	0.8809
Significance				n.s.	n.s.	n.s.

3-Month Male Organ Weights											
Animal Number	Sex	Treatment Group	Time Point	Heart (g)	Thymus (g)	Lungs w/Trachea (g)	Pancreas (g)	Spleen (g)	Kidneys (g)	Liver (g)	Brain (g)
18R0184	M	Control	3 MONTH	1.797	0.626	3.258	1.539	1.016	3.967	21.289	2.251
18R0190	M	Control	3 MONTH	1.748	0.460	2.310	1.160	0.840	4.250	22.300	2.303
18R0191	M	Control	3 MONTH	1.850	0.540	4.310	1.520	0.930	3.780	24.080	2.180
18R0192	M	Control	3 MONTH	1.932	0.556	2.264	1.134	0.980	3.694	23.538	2.216
18R0193	M	Control	3 MONTH	2.010	0.680	3.690	1.700	0.910	4.330	26.860	2.210
Mean				**1.87**	**0.57**	**3.17**	**1.41**	**0.94**	**4.00**	**23.61**	**2.23**
Standard deviation				**0.10**	**0.08**	**0.89**	**0.25**	**0.07**	**0.28**	**2.11**	**0.05**
18R0180	M	DTVRF-AAV5-MAK	3 MONTH	1.889	0.647	2.710	1.202	1.021	4.366	23.037	2.189
18R0181	M	DTVRF-AAV5-MAK	3 MONTH	1.630	0.570	3.220	1.430	1.160	4.350	20.800	2.200
18R0182	M	DTVRF-AAV5-MAK	3 MONTH	1.675	0.519	2.124	1.277	0.858	4.713	22.594	2.260
18R0183	M	DTVRF-AAV5-MAK	3 MONTH	1.690	-	4.340	-	1.270	4.750	22.570	2.160
18R0194	M	DTVRF-AAV5-MAK	3 MONTH	1.880	0.380	2.440	1.330	1.011	4.860	24.800	2.010
Mean				**1.75**	**0.53**	**2.97**	**1.31**	**1.06**	**4.61**	**22.76**	**2.16**
Standard deviation				**0.12**	**0.11**	**0.87**	**0.10**	**0.16**	**0.23**	**1.43**	**0.09**
*P*-value				0.5676	0.5843	0.0796	0.9241	0.2505	0.0151	0.8851	0.6585
Significance				**n.s**.	**n.s**.	**n.s**.	**n.s**.	**n.s**.	*	**n.s**.	**n.s**.

3-Month Female Organ Weights											
Animal Number	Sex	Treatment Group	Time Point	Heart (g)	Thymus (g)	Lungs w/Trachea (g)	Pancreas (g)	Spleen (g)	Kidneys (g)	Liver (g)	Brain (g)
18R0195	F	Control	3 MONTH	1.184	0.675	1.518	0.936	0.685	2.893	15.09	2.181
18R0196	F	Control	3 MONTH	0.970	0.420	2.301	1.060	0.650	2.516	12.520	1.930
18R0197	F	Control	3 MONTH	1.280	0.340	2.430	0.770	0.630	3.000	13.000	2.120
18R0198	F	Control	3 MONTH	1.165	0.365	1.364	1.395	0.561	2.438	11.604	2.030
18R0199	F	Control	3 MONTH	1.260	0.440	2.450	1.120	0.750	2.660	11.800	2.110
Mean				**1.17**	**0.45**	**2.01**	**1.06**	**0.66**	**2.70**	**12.80**	**2.07**
Standard deviation				**0.12**	**0.13**	**0.53**	**0.23**	**0.07**	**0.24**	**1.40**	**0.10**
18R0185	F	DTVRF-AAV5-MAK	3 MONTH	1.001	0.635	1.404	0.424	0.763	2.459	11.135	2.041
18R0186	F	DTVRF-AAV5-MAK	3 MONTH	1.426	0.438	2.331	0.972	0.619	2.567	13.232	2.144
18R0187	F	DTVRF-AAV5-MAK	3 MONTH	1.410	0.420	3.320	1.140	0.790	3.180	16.040	2.090
18R0188	F	DTVRF-AAV5-MAK	3 MONTH	1.292	0.855	1.584	0.557	0.679	2.937	15.358	1.830
18R0189	F	DTVRF-AAV5-MAK	3 MONTH	1.110	0.340	2.290	1.010	0.550	2.410	11.080	2.020
Mean				**1.25**	**0.54**	**2.19**	**0.82**	**0.68**	**2.71**	**13.37**	**2.03**
Standard deviation				**0.19**	**0.21**	**0.76**	**0.31**	**0.10**	**0.33**	**2.31**	**0.12**
*P*-value				0.8193	0.5843	0.0796	0.4418	0.9811	>0.9999	0.9620	0.8358
Significance				**n.s**.	**n.s**.	**n.s**.	**n.s**.	**n.s**.	**n.s**.	**n.s**.	**n.s**.

1-month male organ weights											
Animal number	Sex	Treatment group	Time point	Heart (g)	Thymus (g)	Lungs w/Trachea (g)	Pancreas (g)	Spleen (g)	Kidneys (g)	Liver (g)	Brain (g)
18R0091	M	Control	1 MONTH	1.330	0.940	1.934	2.030	0.900	3.550	18.870	2.780
18R0104	M	Control	1 MONTH	1.380	0.908	2.534	1.153	1.044	3.377	16.932	2.079
18R0105	M	Control	1 MONTH	1.739	0.927	2.129	1.868	1.060	4.208	22.540	2.198
18R0108	M	Control	1 MONTH	1.560	0.680	3.520	1.120	0.810	3.750	18.140	2.390
18R0109	M	Control	1 MONTH	1.670	0.760	2.215	1.650	0.852	3.799	20.670	1.960
Mean				**1.54**	**0.84**	**2.47**	**1.56**	**0.93**	**3.74**	**19.43**	**2.28**
Standard deviation				**0.18**	**0.12**	**0.63**	**0.41**	**0.11**	**0.31**	**2.20**	**0.32**
18R0092	M	DTVRF-AAV5-MAK	1 MONTH	1.430	1.030	1.950	2.770	1.420	3.460	19.850	2.140
18R0095	M	DTVRF-AAV5-MAK	1 MONTH	1.940	0.960	3.000	1.680	0.810	3.910	21.680	2.180
18R0096	M	DTVRF-AAV5-MAK	1 MONTH	1.470	1.140	1.850	2.240	1.005	3.400	21.360	2.050
18R0100	M	DTVRF-AAV5-MAK	1 MONTH	1.948	0.654	2.754	1.181	0.909	3.875	23.250	2.126
18R0101	M	DTVRF-AAV5-MAK	1 MONTH	2.000	1.070	3.170	1.730	0.920	4.220	23.570	2.160
Mean				**1.76**	**0.97**	**2.54**	**1.92**	**1.01**	**3.77**	**21.94**	**2.13**
Standard deviation				**0.28**	**0.19**	**0.61**	**0.61**	**0.24**	**0.34**	**1.51**	**0.05**
*P*-value				0.2312	0.5565	0.1349	0.4839	0.8087	0.9963	0.0757	0.6775
Significance				**n.s**.	**n.s**.	**n.s**.	**n.s**.	**n.s**.	**n.s**.	**n.s**.	**n.s**.

1-month female organ weights											
Animal number	Sex	Treatment group	Time point	Heart (g)	Thymus (g)	Lungs w/Trachea (g)	Pancreas (g)	Spleen (g)	Kidneys (g)	Liver (g)	Brain (g)
18R0098	F	Control	1 MONTH	1.150	0.600	2.520	1.280	0.550	2.350	12.710	2.290
18R0102	F	Control	1 MONTH	1.042	0.570	1.482	1.227	0.517	2.331	12.730	1.568
18R0103	F	Control	1 MONTH	1.220	0.610	2.270	0.980	0.650	2.510	12.800	2.010
18R0106	F	Control	1 MONTH	1.020	0.660	1.970	1.170	0.440	2.030	10.930	1.880
18R0107	F	Control	1 MONTH	0.950	0.854	1.604	0.807	0.522	2.074	10.243	1.894
Mean				**1.08**	**0.66**	**1.97**	**1.09**	**0.54**	**2.26**	**11.88**	**1.93**
Standard deviation				**0.11**	**0.11**	**0.44**	**0.20**	**0.08**	**0.20**	**1.21**	**0.26**
18R0089	F	Vector	1 MONTH	1.009	0.993	2.092	0.765	0.608	2.014	10.303	1.848
18R0090	F	Vector	1 MONTH	1.010	0.730	2.310	1.010	0.540	1.980	11.770	1.940
18R0093	F	Vector	1 MONTH	1.000	0.800	1.970	1.140	0.480	2.240	10.850	1.980
18R0094	F	Vector	1 MONTH	0.945	1.008	1.376	0.871	0.436	2.102	10.243	1.997
18R0097	F	Vector	1 MONTH	1.027	0.606	1.590	1.013	0.547	2.347	11.360	1.890
Mean				**1.00**	**0.83**	**1.87**	**0.96**	**0.52**	**2.14**	**10.91**	**1.93**
Standard deviation				**0.03**	**0.17**	**0.38**	**0.14**	**0.07**	**0.15**	**0.66**	**0.06**
*P*-value				0.8945	0.3280	0.1349	0.9466	0.9987	0.8824	0.7360	>0.9999
Significance				**n.s**.	**n.s**.	**n.s**.	**n.s**.	**n.s**.	**n.s**.	**n.s**.	**n.s**.

3-month male observations					
Animal number	Sex	Treatment group	Time point	Gross observations	Microscopic observations
18R0184	M	Control	3 MONTH	None	Within the liver there is diffuse hepatocellular microvesicular hepatocellular vacuolation. Within the pancreas there are foci of fibrosis which replaces the normal parenchyma and contains a small number of lymphocytes and plasma cells (encircled). There are also multifocal dilated submucosal glands.
18R0190	M	Control	3 MONTH	None	There is a single focus of lymphocytes and plasma cells adjacent to a pancreatic duct. There are also dilated submucosal glands.
18R0191	M	Control	3 MONTH	None	There is a single, small focus of fibrous tissue replacing pancreatic acini intermixed with a low number of lymphocytes and plasma cells. There are also multifocal dilated submucosal glands.
18R0192	M	Control	3 MONTH	None	There is a small focus of neutrophils around a tooth in the periodontal connective tissue. There are rare multifocal, variably sized zones of fibrous tissue replacing pancreatic acini and separating some islet tissues intermixed with a low number of lymphocytes and plasma cells (encircled).
18R0193	M	Control	3 MONTH	None	There is a single focus of purulent inflammation in the periodontal tissue surrounding a single tooth (on right) and a small focus of lymphoplasmacytic inflammation in the harderian gland (left). There are rare multifocal, variably sized zones of fibrous tissue replacing pancreatic acini and separating some islet tissues intermixed with a low number of lymphocytes and plasma cells. There are also multifocal dilated submucosal glands.
18R0180	M	Vector	3 MONTH	None	There is a small focus of periodontal inflammation composed primarily of neutrophils around a molar tooth. There is mild, diffuse microvesicular hepatocellular vacuolation in the liver.
18R0181	M	Vector	3 MONTH	None	There is mild, diffuse microvesicular hepatocellular vacuolation in the liver. Within the renal interstitium there is a single, small focus of lymphocytes and plasma cells. There are also multifocal dilated submucosal glands.
18R0182	M	Vector	3 MONTH	None	There is mild, diffuse microvesicular hepatocellular vacuolation in the liver. Within the renal interstitium there is a single, small focus of lymphocytes and plasma cells.
18R0183	M	Vector	3 MONTH	None	None
18R0194	M	Vector	3 MONTH	None	There is bilateral purulent inflammation in the periodontal tissue surrounding a two of the molar teeth.

3-month female observations					
Animal number	Sex	Treatment group	Time point	Gross observations	Microscopic observations
18R0195	F	Control	3 MONTH	None	There are multifocal dilated submucosal glands within the trachea. Within the kidney at the corticomedullary junction there are mineralized tubules. There are also multifocal dilated submucosal glands.
18R0196	F	Control	3 MONTH	None	Within the skeletal muscle there are small multifocal zones of lymphoplasmacytic inflammation which separate myofibers. Within the pancreas there are multifocal, small zones of pancreatic fibrosis which separate pancreatic acini. There are multifocal dilated submucosal glands within the trachea. Within the kidney at the corticomedullary junction there are mineralized tubules. There are also multifocal dilated submucosal glands.
18R0197	F	Control	3 MONTH	None	There is a moderately sized pocket with viable and degenerative neutrophils surrounded by numerous plasma cells and fewer lymphocytes (abscess) under a molar tooth (encircled). There are multifocal dilated submucosal glands within the trachea. There are also dilated submucosal glands.
18R0198	F	Control	3 MONTH	None	There are dilated submucosal glands.
18R0199	F	Control	3 MONTH	None	None
18R0185	F	Vector	3 MONTH	None	Within the pancreas there are two foci of fibrosis which replace the normal parenchyma and contain a small number of lymphocytes and plasma cells. There are multifocal tubules mineralized at the level of the corticomedullary junction. There are also multifocal dilated submucosal glands.
18R0186	F	Vector	3 MONTH	None	There is mild diffuse microvesicular hepatocellular vacuolation in the liver. There are multifocal tubules mineralized at the level of the corticomedullary junction. There are also multifocal dilated submucosal glands.
18R0187	F	Vector	3 MONTH	None	There is mild diffuse microvesicular hepatocellular vacuolation in the liver. There is a single focus of lymphocytes and plasma cells within the pancreatic intestinum (encircled). There are multifocal tubules mineralized at the level of the corticomedullary junction. There are also multifocal dilated submucosal glands.
18R0188	F	Vector	3 MONTH	None	There is mild diffuse microvesicular hepatocellular vacuolation in the liver. There is a single focus of lymphocytes and plasma cells within the pancreatic intestinum (encircled). There are multifocal tubules mineralized at the level of the corticomedullary junction. There are also multifocal dilated submucosal glands.
18R0189	F	Vector	3 MONTH	None	There are multifocal tubules mineralized at the level of the corticomedullary junction. There are also dilated submucosal glands.

1-month male observations					
Animal number	Sex	Treatment group	Time point	Gross observations	Microscopic observations
18R0091	M	Control	1 MONTH	None	Both liver and spleen have multifocal but rare extramedullary hematopoiesis (EMH). In the liver there is mild microvesicular hepatocellular vacuolation primarily in hepatocytes in the portal triads. There are also multifocal dilated submucosal glands.
18R0104	M	Control	1 MONTH	None	Both liver and spleen have multifocal but rare extramedullary hematopoiesis (EMH). In the liver there is mild microvesicular hepatocellular vacuolation primarily in hepatocytes in the portal triads. There are also multifocal dilated submucosal glands.
18R0105	M	Control	1 MONTH	None	Both liver and spleen have multifocal but rare extramedullary hematopoiesis (EMH). In the liver there is mild microvesicular hepatocellular vacuolation primarily in hepatocytes in the portal triads.
18R0108	M	Control	1 MONTH	None	There is mild, multifocal extramedullary hematopoiesis in the liver and spleen. There are multifocal dilated submucosal glands.
18R0109	M	Control	1 MONTH	None	Rarely, within both harderian glands there are small, multifocal zones of lymphocytes and plasma cells. There is mild, multifocal extramedullary hematopoiesis in the liver and spleen. There are also multifocal dilated submucosal glands.
18R0092	M	Vector	1 MONTH	None	Both liver and spleen have multifocal but rare extramedullary hematopoiesis (EMH). In the liver there is mild microvesicular hepatocellular vacuolation primarily in hepatocytes in the portal triads.
18R0095	M	Vector	1 MONTH	None	Both liver and spleen have multifocal but rare extramedullary hematopoiesis (EMH). In the liver there is mild microvesicular hepatocellular vacuolation primarily in hepatocytes in the portal triads. There are also multifocal dilated submucosal glands.
18R0096	M	Vector	1 MONTH	None	Both liver and spleen have multifocal but rare extramedullary hematopoiesis (EMH). In the liver there is mild microvesicular hepatocellular vacuolation primarily in hepatocytes in the portal triads. There are also multifocal dilated submucosal glands.
18R0100	M	Vector	1 MONTH	None	Both liver and spleen have multifocal but rare extramedullary hematopoiesis (EMH). In the liver there is mild microvesicular hepatocellular vacuolation primarily in hepatocytes in the portal triads.There are also multifocal dilated submucosal glands in the larynx.
18R0101	M	Vector	1 MONTH	None	Both liver and spleen have multifocal but rare extramedullary hematopoiesis (EMH). In the liver there is mild microvesicular hepatocellular vacuolation primarily in hepatocytes in the portal triads. There is a single, moderately sized focus of lymphocytes and fewer plasma cells within the renal interstitium.

1-Month Female Observations					
Animal number	Sex	Treatment group	Time point	Gross observations	Microscopic observations
18R0098	F	Control	1 MONTH	None	Both liver and spleen have multifocal but rare extramedullary hematopoiesis (EMH). In the liver there is mild microvesicular hepatocellular vacuolation primarily in hepatocytes in the portal triads.
18R0102	F	Control	1 MONTH	None	Both liver and spleen have multifocal but rare extramedullary hematopoiesis (EMH). In the liver there is mild microvesicular hepatocellular vacuolation primarily in hepatocytes in the portal triads. Within the thymus there are increased tingible body macrophages (likely indicative of early involution). Within the heart there is a small focus of lymphocytes with fewer plasma cells near the epicardial surface of the left ventricle.
18R0103	F	Control	1 MONTH	None	Both liver and spleen have multifocal but rare extramedullary hematopoiesis (EMH). In the liver there is mild microvesicular hepatocellular vacuolation primarily in hepatocytes in the portal triads. Within the kidney there are multifocal mineralized tubules at the corticomedullary junction. There are also multifocal dilated submucosal glands within the larynx.
18R0106	F	Control	1 MONTH	None	Within the kidney there are multifocal mineralized tubules at the corticomedullary junction.
18R0107	F	Control	1 MONTH	None	There is mild, multifocal extramedullary hematopoiesis in the liver and spleen. Within the trachea there are multifocal dilated submucosal glands. Within the kidney there are multifocal mineralized tubules at the corticomedullary junction. There are also multifocal dilated submucosal glands.
18R0089	F	Vector	1 MONTH	None	Both liver and spleen have multifocal but rare extramedullary hematopoiesis (EMH). In the liver there is mild microvesicular hepatocellular vacuolation primarily in hepatocytes in the portal triads. At the level of the corticomedullary junction there are multifocal mineralized renal tubules. In the thymus there appear to be an increased number of tingible body macrophages within the cortex (could be due to thymic involution which can occur in adult rats normally).
18R0090	F	Vector	1 MONTH	None	Both liver and spleen have multifocal but rare extramedullary hematopoiesis (EMH). In the liver there is mild microvesicular hepatocellular vacuolation primarily in hepatocytes in the portal triads. There are also multifocal dilated submucosal glands.
18R0093	F	Vector	1 MONTH	None	Both liver and spleen have multifocal but rare extramedullary hematopoiesis (EMH). In the liver there is mild microvesicular hepatocellular vacuolation primarily in hepatocytes in the portal triads. There are rare, small foci of lymphocytes and fewer plasma cells within the pancreatic interstitium. Within the peripancreatic fat there are multifocal, small foci of macrophages with fewer lymphocytes and mast cells.
18R0094	F	Vector	1 MONTH	Uterus is slightly fluid-filled (clear).	There is a single focus of lymphocytes and plasma cells with fewer macrophages in the conjunctiva unilaterally (left). Both liver and spleen have multifocal but rare extramedullary hematopoiesis (EMH). In the liver there is mild microvesicular hepatocellular vacuolation primarily in hepatocytes in the portal triads. The uterus diffusely appears dilated but appears empty. There are increased tingible body macrophages multifocally in the thymus. At the level of the corticomedullary junction there are moderate numbers of mineralized tubules. Multifocal submucosal glands are dilated.
18R0097	F	Vector	1 MONTH	None	Both liver and spleen have multifocal but rare extramedullary hematopoiesis (EMH). In the liver there is mild microvesicular hepatocellular vacuolation primarily in hepatocytes in the portal triads.

To further demonstrate systemic tolerability, hematology, and clinical chemistry analysis were also performed for the 40 animals sacrificed at 1-month and 3-month post-injection, in addition to another set of 20 animals (10 M and 10 F, injected in the same manner as above) at a 3-day post-injection time point. At the time of sacrifice, 2 ml of blood was collected from every animal. One milliliter was used for hematology and the remainder was centrifuged for serum collection and clinical chemistry. The hematology and clinical chemistry parameters listed in Supplemental Table 2 were evaluated. As shown in Table 2, no significant AAV5-CMV-*MAK*^*RI*^ induced events were detected between treated and vehicle control animals at any of the timepoints tested for any of the hematology or clinical chemistry parameters evaluated. Collectively, these findings indicate that subretinal injection of AAV5-CMV-*MAK*^*RI*^ (10^11^ vg) is well-tolerated in a mammalian model.

**Table 2:** Clinical chemistry and hematology analysis.

Animal number	18R0184	18R0190	18R0191	18R0192	18R0193	AVG	STD DEV
Sex	M	M	M	M	M	M	M
Time point	3 M	3 M	3 M	3 M	3 M	3 M	3 M
Treatment group	Control	Control	Control	Control	Control	Control	Control
RBC (M/uL)	9.57	8.25	9.33	8.72	8.45	**8.86**	**0.57**
HGB (g/dL)	15.40	14.30	16.30	16.00	14.40	**15.28**	**0.91**
HCT (%)	48.70	45.70	53.00	49.50	45.70	**48.52**	**3.04**
MCV (fL)	50.90	55.40	56.80	56.80	54.10	**54.80**	**2.45**
MCH (pg)	16.10	17.30	17.50	18.30	17.00	**17.24**	**0.80**
MCHC (g/dL)	31.60	31.30	30.80	32.30	31.50	**31.50**	**0.54**
RDW-CV (%)	24.80	21.20	22.00	21.20	21.90	**22.22**	**1.49**
RET (K/uL)	280.40	325.90	358.30	237.20	267.90	**293.94**	**48.07**
RET (%)	2.93	3.95	3.84	2.72	3.17	**3.32**	**0.55**
PLT (K/uL)	238.00	204.00	248.00	123.00	180.00	**198.60**	**50.21**
WBC (K/uL)	14.76	20.46	18.08	16.16	17.62	**17.42**	**2.14**
NEUT (K/uL)	4.10	6.91	4.51	6.52	6.86	**5.78**	**1.36**
NEUT (%)	27.80	33.70	25.00	40.30	38.90	**33.14**	**6.70**
LYMPH (K/uL)	9.58	12.44	12.50	8.61	10.00	**10.63**	**1.76**
LYMPH (%)	64.90	60.80	69.10	53.30	56.80	**60.98**	**6.28**
MONO (K/uL)	0.64	0.65	0.92	0.51	0.64	**0.67**	**0.15**
MONO (%)	4.30	3.20	5.10	3.20	3.60	**3.88**	**0.82**
EO (K/uL)	0.27	0.22	0.15	0.11	0.11	**0.17**	**0.07**
EO (%)	1.80	1.10	0.80	0.70	0.60	**1.00**	**0.48**
BASO (K/uL)	0.17	0.24	0.00	0.41	0.01	**0.17**	**0.17**
BASO (%)	1.20	1.20	0.00	2.50	0.10	**1.00**	**1.02**

Animal number	18R0180	18R0181	18R0182	18R0183	18R0194	AVG	STD DEV	*P*-value	Sig.
Sex	M	M	M	M	M	M	M	M	M
Time point	3 M	3 M	3 M	3 M	3 M	3 M	3 M	3 M	3 M
Treatment group	DTVRF-AAV5-MAK	DTVRF-AAV5-MAK	DTVRF-AAV5-MAK	DTVRF-AAV5-MAK	DTVRF-AAV5-MAK	DTVRF-AAV5-MAK	DTVRF-AAV5-MAK	Control vs. DTVRF-AAV5-MAK	Control vs. DTVRF-AAV5-MAK
RBC (M/uL)	9.47	9.59	9.31	8.99	8.53	**9.18**	**0.43**	0.7414	**n.s**.
HGB (g/dL)	16.00	16.90	16.40	15.00	14.70	**15.80**	**0.93**	0.1919	**n.s**.
HCT (%)	51.90	54.40	53.40	49.50	47.60	**51.36**	**2.80**	0.0665	**n.s**.
MCV (fL)	54.80	56.70	57.40	55.10	55.80	**55.96**	**1.09**	0.6969	**n.s**.
MCH (pg)	16.90	17.60	17.60	16.70	17.20	**17.20**	**0.41**	0.9995	**n.s**.
MCHC (g/dL)	30.80	31.10	30.70	30.30	30.90	**30.76**	**0.30**	0.0488	*
RDW-CV (%)	22.60	22.10	20.90	21.90	20.80	**21.66**	**0.78**	0.8766	**n.s**.
RET (K/uL)	317.20	289.60	284.90	321.80	331.80	**309.06**	**20.66**	0.8722	**n.s**.
RET (%)	3.35	3.02	3.06	3.58	3.89	**3.38**	**0.37**	0.3089	**n.s**.
PLT (K/uL)	266.00	290.00	340.00	872.00	285.00	**410.60**	**259.38**	0.3793	**n.s**.
WBC (K/uL)	16.88	21.50	13.36	12.43	13.73	**15.58**	**3.71**	0.7806	**n.s**.
NEUT (K/uL)	6.00	5.32	5.80	0.48	5.88	**4.70**	**2.37**	0.6392	**n.s**.
NEUT (%)	35.60	24.80	43.50	3.90	42.80	**30.12**	**16.47**	0.9703	**n.s**.
LYMPH (K/uL)	10.13	15.16	7.24	10.78	7.24	**10.11**	**3.26**	0.9891	**n.s**.
LYMPH (%)	60.00	70.50	54.20	86.70	52.70	**64.82**	**14.09**	0.9121	**n.s**.
MONO (K/uL)	0.36	0.84	0.22	0.92	0.34	**0.54**	**0.32**	0.2616	**n.s**.
MONO (%)	2.10	3.90	1.60	7.40	2.50	**3.50**	**2.34**	0.6770	**n.s**.
EO (K/uL)	0.18	0.17	0.10	0.19	0.07	**0.14**	**0.05**	0.8499	**n.s**.
EO (%)	1.10	0.80	0.70	1.50	0.50	**0.92**	**0.39**	0.9871	**n.s**.
BASO (K/uL)	0.21	0.01	0.00	0.06	0.20	**0.10**	**0.10**	0.4375	**n.s**.
BASO (%)	1.20	0.00	0.00	0.50	1.50	**0.64**	**0.69**	0.6234	**n.s**.

Animal number	18R0195	18R0196	18R0197	18R0198	18R0199	AVG	STD DEV
Sex	F	F	F	F	F	F	F
Time point	3 M	3 M	3 M	3 M	3 M	3 M	3 M
Treatment group	Control	Control	Control	Control	Control	Control	Control
RBC (M/uL)	7.76	8.12	8.33	8.26	8.64	**8.22**	**0.32**
HGB (g/dL)	14.20	15.00	15.00	14.90	15.50	**14.92**	**0.47**
HCT (%)	44.80	47.30	48.60	48.30	49.40	**47.68**	**1.78**
MCV (fL)	57.70	58.30	58.30	58.50	57.20	**58.00**	**0.54**
MCH (pg)	18.30	18.50	18.00	18.00	17.90	**18.14**	**0.25**
MCHC (g/dL)	31.70	31.70	30.90	30.80	31.40	**31.30**	**0.43**
RDW-CV (%)	17.30	18.30	18.50	17.20	19.80	**18.22**	**1.06**
RET (K/uL)	251.40	237.90	259.10	252.80	207.40	**241.72**	**20.68**
RET (%)	3.24	2.93	3.11	3.06	2.40	**2.95**	**0.33**
PLT (K/uL)	370.00	526.00	903.00	624.00	878.00	**660.20**	**229.09**
WBC (K/uL)	4.21	6.75	8.29	3.95	9.25	**6.49**	**2.38**
NEUT (K/uL)	0.68	2.38	0.72	0.95	0.59	**1.06**	**0.75**
NEUT (%)	16.10	35.30	8.70	24.10	6.40	**18.12**	**11.84**
LYMPH (K/uL)	3.33	4.20	6.99	2.69	7.76	**4.99**	**2.26**
LYMPH (%)	79.10	62.20	84.30	68.10	83.90	**75.52**	**9.91**
MONO (K/uL)	0.16	0.12	0.47	0.15	0.75	**0.33**	**0.27**
MONO (%)	3.80	1.80	5.70	3.80	8.10	**4.64**	**2.38**
EO (K/uL)	0.04	0.05	0.10	0.02	0.11	**0.06**	**0.04**
EO (%)	1.00	0.70	1.20	0.50	1.20	**0.92**	**0.31**
BASO (K/uL)	0.00	0.00	0.01	0.14	0.04	**0.04**	**0.06**
BASO (%)	0.00	0.00	0.10	3.50	0.40	**0.80**	**1.52**

Animal Number	18R0185	18R0186	18R0187	18R0188	18R0189	AVG	STD DEV	P-value	Sig.
Sex	F	F	F	F	F	F	F	F	F
Time Point	3 M	3 M	3 M	3 M	3 M	3 M	3 M	3 M	3 M
Treatment Group	DTVRF-AAV5-MAK	DTVRF-AAV5-MAK	DTVRF-AAV5-MAK	DTVRF-AAV5-MAK	DTVRF-AAV5-MAK	DTVRF-AAV5-MAK	DTVRF-AAV5-MAK	Control vs. DTVRF-AAV5-MAK	Control vs. DTVRF-AAV5-MAK
RBC (M/uL)	7.87	8.72	8.67	7.31	8.17	**8.15**	**0.59**	0.9949	**n.s**.
HGB (g/dL)	13.80	15.40	15.50	14.00	14.90	**14.72**	**0.79**	0.1919	**n.s**.
HCT (%)	43.40	48.70	49.60	43.80	47.60	**46.62**	**2.85**	0.0665	**n.s**.
MCV (fL)	55.10	55.80	57.20	59.90	58.30	**57.26**	**1.93**	0.8959	**n.s**.
MCH (pg)	17.50	17.70	17.90	19.20	18.20	**18.10**	**0.67**	0.9995	**n.s**.
MCHC (g/dL)	31.80	31.60	31.30	32.00	31.30	**31.60**	**0.31**	0.6563	**n.s**.
RDW-CV (%)	18.10	20.20	19.40	16.90	19.30	**18.78**	**1.29**	0.8766	**n.s**.
RET (K/uL)	281.70	242.40	239.30	207.60	268.00	**247.80**	**28.61**	0.9898	**n.s**.
RET (%)	3.58	2.78	2.76	2.84	3.28	**3.05**	**0.37**	0.3089	**n.s**.
PLT (K/uL)	715.00	786.00	1047.00	936.00	1229.00	**942.60**	**205.73**	0.1651	**n.s**.
WBC (K/uL)	2.93	9.25	11.87	6.04	10.94	**8.21**	**3.69**	0.8127	**n.s**.
NEUT (K/uL)	0.59	1.16	1.40	0.90	1.77	**1.16**	**0.45**	0.9995	**n.s**.
NEUT (%)	20.10	12.50	11.80	14.90	16.20	**15.10**	**3.31**	0.9703	**n.s**.
LYMPH (K/uL)	2.22	7.54	9.50	4.90	8.36	**6.50**	**2.93**	0.7986	**n.s**.
LYMPH (%)	75.80	81.50	80.00	81.10	76.40	**78.96**	**2.68**	0.9345	**n.s**.
MONO (K/uL)	0.10	0.46	0.85	0.19	0.57	**0.43**	**0.30**	0.2616	**n.s**.
MONO (%)	3.40	5.00	7.20	3.10	5.20	**4.78**	**1.64**	0.6770	**n.s**.
EO (K/uL)	0.02	0.08	0.10	0.04	0.19	**0.09**	**0.07**	0.9330	**n.s**.
EO (%)	0.70	0.90	0.80	0.70	1.70	**0.96**	**0.42**	0.9871	**n.s**.
BASO (K/uL)	0.00	0.01	0.02	0.01	0.05	**0.02**	**0.02**	0.4375	**n.s**.
BASO (%)	0.00	0.10	0.20	0.20	0.50	**0.20**	**0.19**	0.6234	**n.s**.

Animal Number	18R0091	18R0104	18R0105	18R0108	18R0109	AVG	STD DEV
Sex	M	M	M	M	M	M	M
Time Point	1 M	1 M	1 M	1 M	1 M	1 M	1 M
Treatment Group	Control	Control	Control	Control	Control	Control	Control
RBC (M/uL)	7.09	8.90	7.56	7.87	7.68	**7.82**	**0.67**
HGB (g/dL)	14.70	16.90	14.80	15.80	14.70	**15.38**	**0.97**
HCT (%)	47.10	53.70	47.10	49.70	45.90	**48.70**	**3.12**
MCV (fL)	66.40	60.30	62.30	63.20	59.80	**62.40**	**2.64**
MCH (pg)	20.70	19.00	19.60	20.10	19.10	**19.70**	**0.71**
MCHC (g/dL)	31.20	31.50	31.40	31.80	32.00	**31.58**	**0.32**
RDW-CV (%)	14.80	17.60	15.90	15.90	15.50	**15.94**	**1.03**
RET (K/uL)	316.20	333.80	336.40	262.10	266.50	**303.00**	**36.21**
RET (%)	4.46	3.75	4.45	3.33	3.47	**3.89**	**0.54**
PLT (K/uL)	126.00	87.00	187.00	178.00	135.00	**142.60**	**40.77**
WBC (K/uL)	5.79	12.70	11.03	12.79	10.30	**10.52**	**2.85**
NEUT (K/uL)	1.13	1.28	6.01	1.45	1.14	**2.20**	**2.13**
NEUT (%)	19.50	10.10	54.40	11.30	11.10	**21.28**	**18.90**
LYMPH (K/uL)	4.37	10.79	4.41	10.58	8.48	**7.73**	**3.18**
LYMPH (%)	75.50	85.00	40.00	82.70	82.30	**73.10**	**18.84**
MONO (K/uL)	0.15	0.40	0.24	0.54	0.53	**0.37**	**0.17**
MONO (%)	2.60	3.10	2.20	4.20	5.10	**3.44**	**1.19**
EO (K/uL)	0.02	0.14	0.04	0.06	0.13	**0.08**	**0.05**
EO (%)	0.30	1.10	0.40	0.50	1.30	**0.72**	**0.45**
BASO (K/uL)	0.12	0.09	0.33	0.16	0.02	**0.14**	**0.12**
BASO (%)	2.10	0.70	3.00	1.30	0.20	**1.46**	**1.11**

Animal Number	18R0092	18R0095	18R0096	18R0100	18R0101	AVG	STD DEV	P-value	Sig.
Sex	M	M	M	M	M	M	M	M	M
Time Point	1 M	1 M	1 M	1 M	1 M	1 M	1 M	1 M	1 M
Treatment Group	DTVRF-AAV5-MAK	DTVRF-AAV5-MAK	DTVRF-AAV5-MAK	DTVRF-AAV5-MAK	DTVRF-AAV5-MAK	DTVRF-AAV5-MAK	DTVRF-AAV5-MAK	Control vs. DTVRF-AAV5-MAK	Control vs. DTVRF-AAV5-MAK
RBC (M/uL)	6.92	7.75	7.49	7.69	7.63	**7.50**	**0.34**	0.7285	**n.s**.
HGB (g/dL)	13.40	15.10	14.80	14.50	15.30	**14.62**	**0.75**	0.4773	**n.s**.
HCT (%)	44.30	48.80	48.50	45.00	48.00	**46.92**	**2.11**	0.6864	**n.s**.
MCV (fL)	64.00	63.00	64.80	58.50	62.90	**62.64**	**2.44**	0.4855	**n.s**.
MCH (pg)	19.40	19.50	19.80	18.90	20.10	**19.54**	**0.45**	0.6364	**n.s**.
MCHC (g/dL)	30.20	30.90	30.50	32.20	31.90	**31.14**	**0.87**	0.4251	**n.s**.
RDW-CV (%)	15.40	18.60	16.80	16.90	16.30	**16.80**	**1.17**	0.1822	**n.s**.
RET (K/uL)	409.70	388.30	340.80	279.90	308.30	**345.40**	**54.00**	0.3759	**n.s**.
RET (%)	5.92	5.01	4.55	3.64	4.04	**4.63**	**0.89**	0.2538	**n.s**.
PLT (K/uL)	229.00	275.00	208.00	185.00	125.00	**204.40**	**55.41**	0.0736	**n.s**.
WBC (K/uL)	17.00	26.24	12.90	15.19	22.13	**18.69**	**5.42**	0.0554	**n.s**.
NEUT (K/uL)	5.08	8.07	5.95	2.05	5.17	**5.26**	**2.16**	0.2287	**n.s**.
NEUT (%)	30.00	30.80	46.10	13.50	23.40	**28.76**	**11.92**	0.2180	**n.s**.
LYMPH (K/uL)	10.31	17.09	5.98	12.02	16.17	**12.31**	**4.52**	0.2350	**n.s**.
LYMPH (%)	60.60	65.10	46.40	79.10	73.10	**64.86**	**12.55**	0.2120	**n.s**.
MONO (K/uL)	1.35	0.51	0.51	0.56	0.65	**0.72**	**0.36**	0.0789	**n.s**.
MONO (%)	7.90	1.90	4.00	3.70	2.90	**4.08**	**2.29**	0.5396	**n.s**.
EO (K/uL)	0.10	0.20	0.07	0.17	0.12	**0.13**	**0.05**	0.3740	**n.s**.
EO (%)	0.60	0.80	0.50	1.10	0.50	**0.70**	**0.25**	0.9630	**n.s**.
BASO (K/uL)	0.16	0.37	0.39	0.39	0.02	**0.27**	**0.17**	0.3371	**n.s**.
BASO (%)	0.90	1.40	3.00	2.60	0.10	**1.60**	**1.20**	0.5601	**n.s**.

Animal Number	18R0098	18R0102	18R0103	18R0106	18R0107	AVG	STD DEV
Sex	F	F	F	F	F	F	F
Time Point	1 M	1 M	1 M	1 M	1 M	1 M	1 M
Treatment Group	Control	Control	Control	Control	Control	Control	Control
RBC (M/uL)	8.27	7.13	7.62	7.98	7.77	**7.75**	**0.43**
HGB (g/dL)	16.70	13.80	14.80	15.60	15.20	**15.22**	**1.06**
HCT (%)	53.10	42.10	47.70	48.90	46.90	**47.74**	**3.96**
MCV (fL)	64.20	59.00	62.60	61.30	60.40	**61.50**	**2.00**
MCH (pg)	20.20	19.40	19.40	19.50	19.60	**19.62**	**0.33**
MCHC (g/dL)	31.50	32.80	31.00	31.90	32.40	**31.92**	**0.71**
RDW-CV (%)	14.70	13.80	14.70	17.40	15.40	**15.20**	**1.35**
RET (K/uL)	240.70	223.20	281.90	296.10	180.30	**244.44**	**46.51**
RET (%)	2.91	3.13	3.70	3.71	2.32	**3.15**	**0.58**
PLT (K/uL)	169.00	102.00	122.00	93.00	93.00	**115.80**	**32.01**
WBC (K/uL)	21.35	3.66	14.21	10.12	11.50	**12.17**	**6.43**
NEUT (K/uL)	7.42	0.30	6.36	1.34	0.89	**3.26**	**3.35**
NEUT (%)	34.70	8.20	44.70	13.20	7.70	**21.70**	**16.94**
LYMPH (K/uL)	12.87	3.15	7.15	8.28	10.13	**8.32**	**3.61**
LYMPH (%)	60.30	86.10	50.30	81.80	88.10	**73.32**	**16.98**
MONO (K/uL)	0.57	0.19	0.31	0.38	0.33	**0.36**	**0.14**
MONO (%)	2.70	5.20	2.20	3.80	2.90	**3.36**	**1.18**
EO (K/uL)	0.11	0.02	0.11	0.12	0.11	**0.09**	**0.04**
EO (%)	0.50	0.50	0.80	1.20	1.00	**0.80**	**0.31**
BASO (K/uL)	0.38	0.00	0.28	0.00	0.04	**0.14**	**0.18**
BASO (%)	1.80	0.00	2.00	0.00	0.30	**0.82**	**1.00**

Animal Number	18R0089	18R0090	18R0093	18R0094	18R0097	AVG	STD DEV	P-value	Sig.
Sex	F	F	F	F	F	F	F	F	F
Time Point	1 M	1 M	1 M	1 M	1 M	1 M	1 M	1 M	1 M
Treatment Group	DTVRF-AAV5-MAK	DTVRF-AAV5-MAK	DTVRF-AAV5-MAK	DTVRF-AAV5-MAK	DTVRF-AAV5-MAK	DTVRF-AAV5-MAK	DTVRF-AAV5-MAK	Control vs. DTVRF-AAV5-MAK	Control vs. DTVRF-AAV5-MAK
RBC (M/uL)	7.61	7.63	7.34	7.57	8.37	**7.70**	**0.39**	0.7285	**n.s**.
HGB (g/dL)	15.20	15.90	14.60	15.10	16.10	**15.38**	**0.61**	0.4773	**n.s**.
HCT (%)	48.00	48.50	47.00	48.50	52.80	**48.96**	**2.23**	0.6864	**n.s**.
MCV (fL)	63.10	63.60	64.00	64.10	63.10	**63.58**	**0.48**	0.4855	**n.s**.
MCH (pg)	20.00	20.80	19.90	19.90	19.20	**19.96**	**0.57**	0.6364	**n.s**.
MCHC (g/dL)	31.70	32.80	31.10	31.10	30.50	**31.44**	**0.87**	0.4251	**n.s**.
RDW-CV (%)	15.60	14.90	16.30	16.50	16.20	**15.90**	**0.65**	0.1822	**n.s**.
RET (K/uL)	232.90	244.20	243.70	265.70	244.40	**246.18**	**11.94**	0.9999	**n.s**.
RET (%)	3.06	3.20	3.32	3.51	2.92	**3.20**	**0.23**	0.9993	**n.s**.
PLT (K/uL)	167.00	108.00	213.00	183.00	361.00	**206.40**	**94.51**	0.0736	**n.s**.
WBC (K/uL)	21.41	15.44	18.77	10.49	19.23	**17.07**	**4.25**	0.0554	**n.s**.
NEUT (K/uL)	8.94	4.61	5.78	5.89	7.70	**6.58**	**1.72**	0.1738	**n.s**.
NEUT (%)	41.80	29.80	30.80	56.10	40.00	**39.70**	**10.61**	0.2180	**n.s**.
LYMPH (K/uL)	11.07	10.18	11.58	4.17	10.68	**9.54**	**3.04**	0.2350	**n.s**.
LYMPH (%)	51.70	65.90	61.70	39.80	55.50	**54.92**	**10.07**	0.2120	**n.s**.
MONO (K/uL)	0.40	0.52	0.58	0.21	0.59	**0.46**	**0.16**	0.0789	**n.s**.
MONO (%)	1.90	3.40	3.10	2.00	3.10	**2.70**	**0.70**	0.5396	**n.s**.
EO (K/uL)	0.20	0.12	0.13	0.09	0.05	**0.12**	**0.06**	0.3740	**n.s**.
EO (%)	0.90	0.80	0.70	0.90	0.30	**0.72**	**0.25**	0.9630	**n.s**.
BASO (K/uL)	0.80	0.01	0.70	0.13	0.21	**0.37**	**0.36**	0.3371	**n.s**.
BASO (%)	3.70	0.10	3.70	1.20	1.10	**1.96**	**1.65**	0.5601	**n.s**.

Animal Number	18R0184	18R0190	18R0191	18R0192	18R0193	AVG	ST DEV
Sex	M	M	M	M	M	M	M
Time Point	3 M	3 M	3 M	3 M	3 M	3 M	3 M
Treatment Group	Control	Control	Control	Control	Control	Control	Control
Total Protein (g/dl)	7.10	6.80	6.90	7.10	6.80	**6.94**	**0.15**
Albumin (g/dl)	3.70	3.50	3.50	3.50	3.40	**3.52**	**0.11**
Globulin (g/dl)	3.40	3.30	3.40	3.60	3.40	**3.42**	**0.11**
Sodium (mEq/L)	147.20	145.40	144.20	148.60	144.70	**146.02**	**1.84**
Potassium (mEq/L)	6.60	6.26	9.27	6.36	6.74	**7.05**	**1.26**
Chloride (mEq/L)	97.70	95.40	95.90	97.60	95.70	**96.46**	**1.10**
Total CO2 (mEq/L)	39.00	39.00	33.00	36.00	34.00	**36.20**	**2.77**
Calcium (g/dl)	12.10	11.80	12.50	11.70	12.20	**12.06**	**0.32**
Glucose (mg/dl)	160.00	166.00	231.00	168.00	264.00	**197.80**	**46.94**
Alkaline Phosphatase (U/L)	198.00	295.00	264.00	207.00	190.00	**230.80**	**46.18**
Alanine aminotransferase (U/L)	43.00	49.00	47.00	44.00	46.00	**45.80**	**2.39**
Aspartate aminotransferase (U/L)	95.00	73.00	57.00	59.00	59.00	**68.60**	**16.09**
Lactate dehydrogenase (U/L)	306.00	174.00	128.00	135.00	143.00	**177.20**	**74.11**
Total bilirubin (mg/dl)	0.20	0.20	0.20	0.20	0.10	**0.18**	**0.04**
Phosphorus (mg/dl)	9.60	8.70	10.60	9.50	10.40	**9.76**	**0.76**
Blood urea nitrogen (mg/dl)	14.00	17.00	19.00	21.00	16.00	**17.40**	**2.70**
Creatinine (mg/dl)	0.33	0.33	0.21	0.42	0.33	**0.32**	**0.07**
Cholesterol (mg/dl)	82.00	90.00	75.00	82.00	83.00	**82.40**	**5.32**
Triglycerides (mg/dl)	181.00	223.00	227.00	310.00	117.00	**211.60**	**70.60**
Creatine Kinase (U/L)	364.00	115.00	89.00	87.00	89.00	**148.80**	**120.86**
Uric Acid (mg/dl)	2.03	-	3.73	1.59	2.45	**2.45**	**0.92**
HDL (mg/dl)	29.00	28.00	27.00	26.00	29.00	**27.80**	**1.30**

Animal Number	18R0180	18R0181	18R0182	18R0183	18R0194	AVG	ST DEV	P-value	Sig.
Sex	M	M	M	M	M	M	M	M	M
Time Point	3 M	3 M	3 M	3 M	3 M	3 M	3 M	3 M	3 M
Treatment Group	DTVRF-AAV5-MAK	DTVRF-AAV5-MAK	DTVRF-AAV5-MAK	DTVRF-AAV5-MAK	DTVRF-AAV5-MAK	DTVRF-AAV5-MAK	DTVRF-AAV5-MAK	Control vs. DTVRF-AAV5-MAK	Control vs. DTVRF-AAV5-MAK
Total Protein (g/dl)	7.20	7.10	7.00	6.50	7.10	**6.98**	**0.28**	0.9982	**n.s**.
Albumin (g/dl)	3.70	3.70	3.70	3.50	3.60	**3.64**	**0.09**	0.8589	**n.s**.
Globulin (g/dl)	3.50	3.40	3.30	3.00	3.50	**3.34**	**0.21**	0.4737	**n.s**.
Sodium (mEq/L)	145.20	145.40	149.70	147.20	146.60	**146.82**	**1.81**	0.5768	**n.s**.
Potassium (mEq/L)	7.35	7.20	6.87	6.29	6.03	**6.75**	**0.57**	0.0724	**n.s**.
Chloride (mEq/L)	96.90	96.90	96.10	98.40	94.40	**96.54**	**1.46**	0.4727	**n.s**.
Total CO2 (mEq/L)	34.00	35.00	46.00	39.00	35.00	**37.80**	**4.97**	0.3689	**n.s**.
Calcium (g/dl)	12.30	12.10	11.80	12.10	11.50	**11.96**	**0.31**	0.2572	**n.s**.
Glucose (mg/dl)	178.00	241.00	129.00	166.00	152.00	**173.20**	**42.06**	0.5295	**n.s**.
Alkaline Phosphatase (U/L)	227.00	187.00	195.00	258.00	330.00	**239.40**	**57.93**	0.9932	**n.s**.
Alanine aminotransferase (U/L)	40.00	41.00	36.00	46.00	44.00	**41.40**	**3.85**	0.3837	**n.s**.
Aspartate aminotransferase (U/L)	55.00	53.00	53.00	57.00	71.00	**57.80**	**7.56**	0.7610	**n.s**.
Lactate dehydrogenase (U/L)	224.00	160.00	124.00	134.00	270.00	**182.40**	**62.57**	0.9717	**n.s**.
Total bilirubin (mg/dl)	0.20	0.20	0.20	0.10	0.10	**0.16**	**0.05**	0.5847	**n.s**.
Phosphorus (mg/dl)	9.40	9.40	8.90	8.80	8.30	**8.96**	**0.46**	0.4652	**n.s**.
Blood urea nitrogen (mg/dl)	17.00	16.00	15.00	18.00	15.00	**16.20**	**1.30**	0.4256	**n.s**.
Creatinine (mg/dl)	0.31	0.32	0.35	0.38	0.29	**0.33**	**0.04**	0.8783	**n.s**.
Cholesterol (mg/dl)	87.00	90.00	105.00	78.00	78.00	**87.60**	**11.10**	0.2731	**n.s**.
Triglycerides (mg/dl)	181.00	187.00	176.00	129.00	124.00	**159.40**	**30.34**	0.4882	**n.s**.
Creatine Kinase (U/L)	100.00	99.00	101.00	95.00	145.00	**108.00**	**20.81**	0.6122	**n.s**.
Uric Acid (mg/dl)	2.09	2.88	-	1.71	-	**2.23**	**0.60**	0.9632	**n.s**.
HDL (mg/dl)	25.00	29.00	37.00	26.00	24.00	**28.20**	**5.26**	0.0646	**n.s**.

Animal Number	18R0195	18R0196	18R0197	18R0198	18R0199	AVG	ST DEV
Sex	F	F	F	F	F	F	F
Time Point	3 M	3 M	3 M	3 M	3 M	3 M	3 M
Treatment Group	Control	Control	Control	Control	Control	Control	Control
Total Protein (g/dl)	8.20	7.90	8.50	7.70	7.40	**7.94**	**0.43**
Albumin (g/dl)	4.70	4.30	4.80	4.20	4.10	**4.42**	**0.31**
Globulin (g/dl)	3.50	3.60	3.70	3.50	3.30	**3.52**	**0.15**
Sodium (mEq/L)	146.90	146.30	147.50	146.70	144.40	**146.36**	**1.18**
Potassium (mEq/L)	5.22	5.72	4.87	5.86	6.06	**5.55**	**0.49**
Chloride (mEq/L)	97.90	96.00	96.00	97.60	96.20	**96.74**	**0.93**
Total CO2 (mEq/L)	38.00	37.00	29.00	33.00	32.00	**33.80**	**3.70**
Calcium (g/dl)	12.90	11.70	13.10	12.50	12.50	**12.54**	**0.54**
Glucose (mg/dl)	116.00	128.00	195.00	119.00	230.00	**157.60**	**51.81**
Alkaline Phosphatase (U/L)	213.00	110.00	123.00	108.00	56.00	**122.00**	**56.96**
Alanine aminotransferase (U/L)	33.00	34.00	53.00	53.00	36.00	**41.80**	**10.28**
Aspartate aminotransferase (U/L)	84.00	68.00	60.00	94.00	47.00	**70.60**	**18.73**
Lactate dehydrogenase (U/L)	213.00	344.00	62.00	237.00	121.00	**195.40**	**108.92**
Total bilirubin (mg/dl)	0.10	0.20	0.10	0.20	0.10	**0.14**	**0.05**
Phosphorus (mg/dl)	8.30	7.50	7.90	10.60	9.90	**8.84**	**1.34**
Blood urea nitrogen (mg/dl)	17.00	17.00	16.00	16.00	17.00	**16.60**	**0.55**
Creatinine (mg/dl)	0.36	0.38	0.33	0.31	0.34	**0.34**	**0.03**
Cholesterol (mg/dl)	134.00	81.00	122.00	94.00	78.00	**101.80**	**25.02**
Triglycerides (mg/dl)	140.00	224.00	122.00	125.00	147.00	**151.60**	**41.78**
Creatine Kinase (U/L)	376.00	173.00	112.00	375.00	67.00	**220.60**	**146.32**
Uric Acid (mg/dl)	-	-	1.70	2.00	2.95	**2.22**	**0.65**
HDL (mg/dl)	46.00	30.00	42.00	35.00	28.00	**36.20**	**7.69**

Animal Number	18R0185	18R0186	18R0187	18R0188	18R0189	AVG	ST DEV	P-value	Sig.
Sex	F	F	F	F	F	F	F	F	F
Time Point	3 M	3 M	3 M	3 M	3 M	3 M	3 M	3 M	3 M
Treatment Group	DTVRF-AAV5-MAK	DTVRF-AAV5-MAK	DTVRF-AAV5-MAK	DTVRF-AAV5-MAK	DTVRF-AAV5-MAK	DTVRF-AAV5-MAK	DTVRF-AAV5-MAK	Control vs. DTVRF-AAV5-MAK	Control vs. DTVRF-AAV5-MAK
Total Protein (g/dl)	7.50	7.30	8.60	7.40	7.50	**7.66**	**0.53**	0.6493	**n.s**.
Albumin (g/dl)	4.00	4.10	4.80	4.10	4.00	**4.20**	**0.34**	0.4916	**n.s**.
Globulin (g/dl)	3.50	3.20	3.80	3.30	3.50	**3.46**	**0.23**	0.4737	**n.s**.
Sodium (mEq/L)	146.20	144.30	146.00	145.50	145.70	**145.54**	**0.74**	0.5768	**n.s**.
Potassium (mEq/L)	7.72	6.42	7.09	5.40	5.93	**6.51**	**0.92**	0.0724	**n.s**.
Chloride (mEq/L)	100.00	96.50	97.20	96.10	98.40	**97.64**	**1.58**	0.4727	**n.s**.
Total CO2 (mEq/L)	38.00	36.00	31.00	36.00	33.00	**34.80**	**2.77**	0.3689	**n.s**.
Calcium (g/dl)	12.30	11.80	13.60	12.00	12.20	**12.38**	**0.71**	0.2572	**n.s**.
Glucose (mg/dl)	134.00	156.00	205.00	150.00	195.00	**168.00**	**30.50**	0.5295	**n.s**.
Alkaline Phosphatase (U/L)	90.00	171.00	78.00	91.00	151.00	**116.20**	**41.82**	0.9979	**n.s**.
Alanine aminotransferase (U/L)	39.00	52.00	65.00	52.00	38.00	**49.20**	**11.12**	0.3837	**n.s**.
Aspartate aminotransferase (U/L)	132.00	59.00	48.00	54.00	58.00	**70.20**	**34.82**	0.7610	**n.s**.
Lactate dehydrogenase (U/L)	332.00	145.00	114.00	157.00	102.00	**170.00**	**93.27**	0.9717	**n.s**.
Total bilirubin (mg/dl)	0.20	0.10	0.20	0.10	0.10	**0.14**	**0.05**	0.5847	**n.s**.
Phosphorus (mg/dl)	11.20	8.50	9.90	9.30	8.20	**9.42**	**1.20**	0.4652	**n.s**.
Blood urea nitrogen (mg/dl)	16.00	14.00	17.00	14.00	17.00	**15.60**	**1.52**	0.4256	**n.s**.
Creatinine (mg/dl)	0.43	0.29	0.35	0.33	0.33	**0.35**	**0.05**	0.8783	**n.s**.
Cholesterol (mg/dl)	80.00	98.00	109.00	78.00	88.00	**90.60**	**12.95**	0.2731	**n.s**.
Triglycerides (mg/dl)	100.00	120.00	301.00	267.00	110.00	**179.60**	**96.32**	0.4882	**n.s**.
Creatine Kinase (U/L)	758.00	103.00	81.00	133.00	119.00	**238.80**	**290.89**	0.6122	**n.s**.
Uric Acid (mg/dl)	2.28	-	2.53	-	2.20	**2.34**	**0.17**	0.9632	**n.s**.
HDL (mg/dl)	28.00	37.00	35.00	28.00	33.00	**32.20**	**4.09**	0.0646	**n.s**.

Animal Number	18R0091	18R0104	18R0105	18R0108	18R0109	AVG	ST DEV
Sex	M	M	M	M	M	M	M
Time Point	1 M	1 M	1 M	1 M	1 M	1 M	1 M
Treatment Group	Control	Control	Control	Control	Control	Control	Control
Total Protein (g/dl)	6.60	6.70	6.80	7.10	6.50	**6.74**	**0.23**
Albumin (g/dl)	3.50	3.60	3.60	3.70	3.60	**3.60**	**0.07**
Globulin (g/dl)	3.10	3.10	3.20	3.40	2.90	**3.14**	**0.18**
Sodium (mEq/L)	145.00	146.10	148.10	149.40	149.20	**147.56**	**1.94**
Potassium (mEq/L)	6.52	6.01	7.03	5.50	5.41	**6.09**	**0.69**
Chloride (mEq/L)	98.00	97.50	96.80	98.10	97.40	**97.56**	**0.52**
Total CO2 (mEq/L)	32.00	26.00	37.00	33.00	35.00	**32.60**	**4.16**
Calcium (g/dl)	11.90	13.90	12.50	12.80	12.60	**12.74**	**0.73**
Glucose (mg/dl)	126.00	381.00	147.00	228.00	206.00	**217.60**	**100.40**
Alkaline Phosphatase (U/L)	541.00	299.00	336.00	316.00	271.00	**352.60**	**107.98**
Alanine aminotransferase (U/L)	46.00	51.00	56.00	51.00	52.00	**51.20**	**3.56**
Aspartate aminotransferase (U/L)	100.00	62.00	165.00	65.00	74.00	**93.20**	**42.83**
Lactate dehydrogenase (U/L)	355.00	127.00	393.00	182.00	112.00	**233.80**	**131.30**
Total bilirubin (mg/dl)	0.20	0.10	0.10	0.20	0.10	**0.14**	**0.05**
Phosphorus (mg/dl)	10.80	10.40	11.40	10.60	10.80	**10.80**	**0.37**
Blood urea nitrogen (mg/dl)	16.00	16.00	14.00	15.00	15.00	**15.20**	**0.84**
Creatinine (mg/dl)	0.40	0.39	0.33	0.38	0.34	**0.37**	**0.03**
Cholesterol (mg/dl)	75.00	74.00	88.00	92.00	85.00	**82.80**	**7.98**
Triglycerides (mg/dl)	251.00	272.00	170.00	204.00	111.00	**201.60**	**64.44**
Creatine Kinase (U/L)	439.00	127.00	947.00	98.00	246.00	**371.40**	**348.60**
Uric Acid (mg/dl)	2.09	6.00	2.74	2.56	3.06	**3.29**	**1.56**
HDL (mg/dl)	28.00	25.00	33.00	30.00	32.00	**29.60**	**3.21**

Animal Number	18R0092	18R0095	18R0096	18R0100	18R0101	AVG	ST DEV	P-value	Sig.
Sex	M	M	M	M	M	M	M	M	M
Time Point	1 M	1 M	1 M	1 M	1 M	1 M	1 M	1 M	1 M
Treatment Group	DTVRF-AAV5-MAK	DTVRF-AAV5-MAK	DTVRF-AAV5-MAK	DTVRF-AAV5-MAK	DTVRF-AAV5-MAK	DTVRF-AAV5-MAK	DTVRF-AAV5-MAK	Control vs. DTVRF-AAV5-MAK	Control vs. DTVRF-AAV5-MAK
Total Protein (g/dl)	6.40	7.10	6.50	6.60	7.20	6.76	0.36	0.1600	n.s.
Albumin (g/dl)	3.40	3.70	3.50	3.40	3.60	3.52	0.13	0.9251	n.s.
Globulin (g/dl)	3.00	3.40	3.00	3.20	3.60	3.24	0.26	0.1191	n.s.
Sodium (mEq/L)	145.10	146.60	147.90	146.00	148.20	146.76	1.30	0.1188	n.s.
Potassium (mEq/L)	7.30	5.01	5.71	5.89	7.13	6.21	0.98	0.9028	n.s.
Chloride (mEq/L)	98.70	95.20	97.60	95.90	98.60	97.20	1.59	0.9768	n.s.
Total CO2 (mEq/L)	24.00	30.00	34.00	34.00	31.00	30.60	4.10	0.2083	n.s.
Calcium (g/dl)	14.60	13.20	12.60	12.30	13.20	13.18	0.88	0.4275	n.s.
Glucose (mg/dl)	466.00	255.00	156.00	187.00	264.00	265.60	120.90	0.7243	n.s.
Alkaline Phosphatase (U/L)	554.00	274.00	331.00	220.00	305.00	336.80	128.26	0.9918	n.s.
Alanine aminotransferase (U/L)	43.00	39.00	40.00	42.00	42.00	41.20	1.64	0.0895	n.s.
Aspartate aminotransferase (U/L)	100.00	60.00	68.00	51.00	62.00	68.20	18.79	0.6927	n.s.
Lactate dehydrogenase (U/L)	202.00	168.00	126.00	130.00	179.00	161.00	32.56	0.7072	n.s.
Total bilirubin (mg/dl)	0.10	0.10	0.10	0.10	0.20	0.12	0.04	0.2140	n.s.
Phosphorus (mg/dl)	13.50	10.80	11.90	10.60	11.40	11.64	1.16	0.4455	n.s.
Blood urea nitrogen (mg/dl)	17.00	15.00	14.00	18.00	16.00	16.00	1.58	0.3935	n.s.
Creatinine (mg/dl)	0.44	0.38	0.32	0.35	0.37	0.37	0.04	0.9541	n.s.
Cholesterol (mg/dl)	111.00	96.00	85.00	109.00	114.00	103.00	12.19	0.0883	n.s.
Triglycerides (mg/dl)	220.00	148.00	159.00	199.00	172.00	179.60	29.53	0.3095	n.s.
Creatine Kinase (U/L)	377.00	152.00	209.00	105.00	98.00	188.20	114.51	0.6421	n.s.
Uric Acid (mg/dl)	7.98	3.30	3.14	1.35	3.88	3.93	2.45	0.8916	n.s.
HDL (mg/dl)	38.00	40.00	31.00	36.00	38.00	36.60	3.44	0.0552	n.s.

Animal Number	18R0098	18R0102	18R0103	18R0106	18R0107	AVG	ST DEV
Sex	F	F	F	F	F	F	F
Time Point	1 M	1 M	1 M	1 M	1 M	1 M	1 M
Treatment Group	Control	Control	Control	Control	Control	Control	Control
Total Protein (g/dl)	7.20	7.20	7.00	8.30	6.70	**7.28**	**0.61**
Albumin (g/dl)	4.00	4.20	4.10	4.70	3.80	**4.16**	**0.34**
Globulin (g/dl)	3.20	3.00	2.90	3.60	2.90	**3.12**	**0.29**
Sodium (mEq/L)	145.20	147.80	145.10	145.40	143.40	**145.38**	**1.57**
Potassium (mEq/L)	5.52	7.03	6.19	7.02	6.55	**6.46**	**0.63**
Chloride (mEq/L)	96.80	100.90	97.60	96.30	95.70	**97.46**	**2.05**
Total CO2 (mEq/L)	30.00	30.00	26.00	29.00	31.00	**29.20**	**1.92**
Calcium (g/dl)	12.80	12.30	13.40	14.10	12.70	**13.06**	**0.70**
Glucose (mg/dl)	221.00	176.00	312.00	254.00	209.00	**234.40**	**51.58**
Alkaline Phosphatase (U/L)	144.00	190.00	137.00	222.00	216.00	**181.80**	**39.65**
Alanine aminotransferase (U/L)	39.00	65.00	40.00	39.00	37.00	**44.00**	**11.79**
Aspartate aminotransferase (U/L)	62.00	307.00	53.00	52.00	47.00	**104.20**	**113.50**
Lactate dehydrogenase (U/L)	148.00	1184.00	151.00	114.00	97.00	**338.80**	**473.03**
Total bilirubin (mg/dl)	0.10	0.10	0.10	0.10	0.10	**0.10**	**0.00**
Phosphorus (mg/dl)	10.80	11.50	10.10	11.30	11.60	**11.06**	**0.62**
Blood urea nitrogen (mg/dl)	17.00	17.00	15.00	17.00	17.00	**16.60**	**0.89**
Creatinine (mg/dl)	0.36	0.43	0.38	0.40	0.36	**0.39**	**0.03**
Cholesterol (mg/dl)	73.00	89.00	78.00	115.00	79.00	**86.80**	**16.80**
Triglycerides (mg/dl)	56.00	117.00	104.00	184.00	191.00	**130.40**	**56.91**
Creatine Kinase (U/L)	122.00	2458.00	101.00	72.00	102.00	**571.00**	**1055.02**
Uric Acid (mg/dl)	2.53	3.09	4.43	3.51	2.37	**3.19**	**0.83**
HDL (mg/dl)	29.00	33.00	31.00	41.00	31.00	**33.00**	**4.69**

Animal Number	18R0089	18R0090	18R0093	18R0094	18R0097	AVG	ST DEV	P-value	Sig.
Sex	F	F	F	F	F	F	F	F	F
Time Point	1 M	1 M	1 M	1 M	1 M	1 M	1 M	1 M	1 M
Treatment Group	DTVRF-AAV5-MAK	DTVRF-AAV5-MAK	DTVRF-AAV5-MAK	DTVRF-AAV5-MAK	DTVRF-AAV5-MAK	DTVRF-AAV5-MAK	DTVRF-AAV5-MAK	Control vs. DTVRF-AAV5-MAK	Control vs. DTVRF-AAV5-MAK
Total Protein (g/dl)	7.20	7.20	7.00	8.30	6.70	**7.28**	**0.61**	0.1600	**n.s**.
Albumin (g/dl)	4.00	4.20	4.10	4.70	3.80	**4.16**	**0.34**	0.7916	**n.s**.
Globulin (g/dl)	3.20	3.00	2.90	3.60	2.90	**3.12**	**0.29**	0.1191	**n.s**.
Sodium (mEq/L)	145.20	147.80	145.10	145.40	143.40	**145.38**	**1.57**	0.1188	**n.s**.
Potassium (mEq/L)	5.52	7.03	6.19	7.02	6.55	**6.46**	**0.63**	0.9028	**n.s**.
Chloride (mEq/L)	96.80	100.90	97.60	96.30	95.70	**97.46**	**2.05**	0.9768	**n.s**.
Total CO2 (mEq/L)	30.00	30.00	26.00	29.00	31.00	**29.20**	**1.92**	0.2083	**n.s**.
Calcium (g/dl)	12.80	12.30	13.40	14.10	12.70	**13.06**	**0.70**	0.4275	**n.s**.
Glucose (mg/dl)	221.00	176.00	312.00	254.00	209.00	**234.40**	**51.58**	0.7243	**n.s**.
Alkaline Phosphatase (U/L)	144.00	190.00	137.00	222.00	216.00	**181.80**	**39.65**	>0.9999	**n.s**.
Alanine aminotransferase (U/L)	39.00	65.00	40.00	39.00	37.00	**44.00**	**11.79**	0.0895	**n.s**.
Aspartate aminotransferase (U/L)	62.00	307.00	53.00	52.00	47.00	**104.20**	**113.50**	0.6927	**n.s**.
Lactate dehydrogenase (U/L)	148.00	1184.00	151.00	114.00	97.00	**338.80**	**473.03**	0.7072	**n.s**.
Total bilirubin (mg/dl)	0.10	0.10	0.10	0.10	0.10	**0.10**	**0.00**	0.2140	**n.s**.
Phosphorus (mg/dl)	10.80	11.50	10.10	11.30	11.60	**11.06**	**0.62**	0.4455	**n.s**.
Blood urea nitrogen (mg/dl)	17.00	17.00	15.00	17.00	17.00	**16.60**	**0.89**	0.3935	**n.s**.
Creatinine (mg/dl)	0.36	0.43	0.38	0.40	0.36	**0.39**	**0.03**	0.9541	**n.s**.
Cholesterol (mg/dl)	73.00	89.00	78.00	115.00	79.00	**86.80**	**16.80**	0.0883	**n.s**.
Triglycerides (mg/dl)	56.00	117.00	104.00	184.00	191.00	**130.40**	**56.91**	0.3095	**n.s**.
Creatine Kinase (U/L)	122.00	2458.00	101.00	72.00	102.00	**571.00**	**1055.02**	0.6421	**n.s**.
Uric Acid (mg/dl)	2.53	3.09	4.43	3.51	2.37	**3.19**	**0.83**	0.8916	**n.s**.
HDL (mg/dl)	29.00	33.00	31.00	41.00	31.00	**33.00**	**4.69**	0.0552	**n.s**.

## Discussion

Inherited retinal degeneration can be caused by mutations in any of more than 100 different genes [4]. More than 75% of the retinal-disease-causing genes that behave in a recessive fashion [67/84, [4]] are small enough to be packaged into an AAV making them ideal candidates for clinical gene augmentation therapy. Unfortunately, the paucity of model systems that faithfully recapitulate disease phenotypes and the costs associated with manufacturing and testing clinical gene therapy vectors make it challenging to carry a gene therapy candidate from bench to bedside in a rapid cost-effective manner. To make significant progress toward treating all patients with inherited retinal degeneration, the time and cost associated with the development of clinical grade gene-based therapeutics both need to be reduced.

To address the lack of model systems suitable for evaluating treatment efficacy we have focused our attention on the use of patient-iPSC-derived retinal cells. Unlike animal models, which take significant time and money to create, the human iPSC field has evolved to the point where disease-specific cell types can be readily generated, and disease phenotypes evaluated quickly and inexpensively [recently reviewed by Mullin et al. [20]]. In this study, we used iPSCs generated from three independent patients with molecularly-confirmed *MAK*-associated RP as a model system to evaluate AAV-mediated *MAK* gene replacement at the transcriptional, translational, immunocytochemical, and functional levels in human cells.

We previously found that the 75 bp exon 12 of the MAK gene is expressed exclusively in the retina and has been highly conserved since first appearing in reptiles nearly 400 million years ago [1]. Interestingly, both the canonical and retinal-specific versions of the MAK protein are expressed in the human retina [1] and when present in isolation, either isoform has the ability to regulate cilia length, suggesting that the gene’s primary function is independent of exon 12. It was somewhat surprising to find that inclusion of the retina-specific exon in our gene transfer constructs dramatically increased expression of the *MAK* transcript and protein regardless of the strength of the promoter used to drive the gene. This led us to believe initially that exon 12 was acting as a transcriptional enhancer element. However, when equimolar amounts of canonical and retina-specific *MAK* mRNAs were injected directly into developing zebrafish, bypassing the transcriptional machinery, the retina-specific isoform was still expressed more robustly than the canonical one (Supplemental Fig. 2). These findings suggest that exon 12 may play a role in stabilizing the *MAK* transcript or promoting its translation. Regardless of mechanism, it seems likely that the primary evolutionary advantage of *MAK* exon 12 is to ensure robust expression of the protein in photoreceptor cells where integrity of the connecting cilia is critically important for cell viability. Unlike most cell types in which primary cilia are small sensory organelles that account for a minute portion of the cell’s total surface area, the photoreceptor outer segment is a modified primary cilium that makes up the majority of the cell’s mass [21–24]. On average, a rod photoreceptor’s outer segment is completely recycled every ten days requiring massive amount of protein to be shuttled between the photoreceptor inner and outer segment via the connecting cilium [25]. In the absence of efficient protein trafficking, outer segment turnover and phototransduction are disrupted resulting in visual dysfunction. As such, it is not surprising that many of the genes that cause RP [e.g., BBS genes, CEP290, IFT88, etc. [4, 26, 27]] are required for proper protein trafficking through the connecting cilium.

After determining that CMV-driven retinal-*MAK* promoted the greatest degree of phenotypic correction, the next step was to demonstrate the safety of a dose equal to or greater than what would be proposed as the maximum clinical dose in a phase 1 human trial. To calculate a human equivalent dose, one must consider the number of cells being targeted in both the animal model being used and the eventual human patients. Although large animal models with eye sizes similar to humans (e.g., pigs) allow one to deliver exactly the same volume of drug to the subretinal space as would be used in humans, the cost associated with housing a large number of pigs for at least 3 months, which is the current post-injection survival time being requested by the FDA, is prohibitive. In addition, few facilities have the space to maintain more than a handful of large animals at any given time making simultaneous testing of multiple therapeutic vectors challenging. Because they are relatively inexpensive to house, mice have been used extensively for preclinical analysis of novel gene-based therapeutics. Unfortunately, the volume of drug that can be delivered to the subretinal space of the mouse is very small, typically less than 1ul. In addition, the amount of blood that can be obtained following injection for hematology and clinical chemistry analysis is so low that it often requires duplication of study animals, which significantly increases the cost and time needed to perform the study. We have found the normal adult Sprague Dawley rat to be an ideal model system for safety analysis. Sprague Dawley rats are readily available, relatively inexpensive, and have been very well characterized with many strain specific lesions and hematologic findings thoroughly described. At just two months of age, Sprague Dawley rats are large enough that one can reliably inject a dose of AAV into the subretinal space that would be equivalent to the maximum clinical dose delivered in human without needing virus of exceptionally high titer. In addition, rats have significantly more total blood volume than mice, which allows one to perform a complete hematology and blood chemistry panel on each animal. Finally, the Sprague Dawley rat is small enough that it can be housed in sufficient numbers to perform all of the post-injection survival timepoints required for testing multiple products at the same time.

**Fig. 5:**
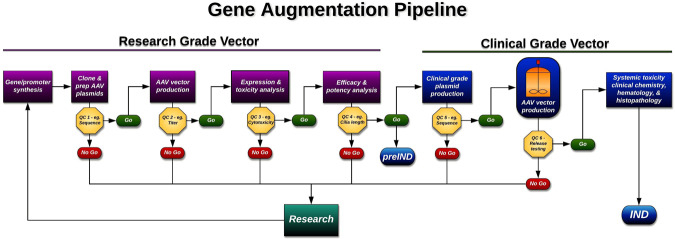
Schematic of therapeutic development pipeline described and followed in this study. Using this strategy we were able to develop, test, and validate enough product to treat 500 patients (at a maximum clinical dose of 1 × 10^11^v.g.) with MAK associated RP for less than $500,000 USD (i.e., ~$1000 USD/patient). v.g. = viral genomes. QC = quality control. Example QC analysis performed at each step is provided. For a complete list of QC analysis performed see Supplemental Table 3.

As indicated above, in this manuscript we report development of a pipeline specifically designed to allow for rapid evaluation of novel gene therapy candidates (Fig. 5). As a gene therapy product progresses from synthesis through to filing of an IND application, the cost associated with each stage within the pipeline increases significantly. We have incorporated several go/no go quality control check points (Table S3), at which the decision to progress to the next stage of production and testing or to return to research and development must be made. To keep cost low, early expression, cytotoxicity, and efficacy testing are done using research grade virus produced in the lab using cGMP compatible protocols and reagents. Once confident that we are able to safely overexpress the gene of interest and in turn restore functional protein in a relevant model system, the next step is to proceed with clinical vector production and detailed toxicity analysis, which is where a majority of the cost associated with this process is incurred. It is at this point where we recommend engaging with the FDA in the form of a pre-IND application and meeting (i.e., to get their opinion on suitability of data in hand and proposed or ongoing preclinical safety testing for initiation of a phase 1/2 safety trial). Following completion of clinical vector release testing and preclinical safety analysis, with the support of FDA consultants data are packaged and submitted to the FDA in an investigator initiated IND application.

One of the greatest misconceptions among patients diagnosed with retinitis pigmentosa is that restoration of functional gene expression will result in restoration of vision. While this is true in the rare circumstance in which the disease is caused by dysfunction of a living cell, for the majority of patients with an inherited retinal disease successful gene augmentation will at best arrest the progression of the disease. The most important factor for successful therapy is likely to be early intervention, before so many rod photoreceptors have been lost that there is an insufficient amount of critical rod-derived retinal viability factors and too high a level of intra-retinal oxygen for the remaining cone photoreceptors to survive long term. *MAK*-associated RP has two favorable avenues for early intervention. First, this disease is a relatively mild form of RP and many patients still have a large number of photoreceptors when they are first diagnosed in early adulthood. Second, the Jewish population is more likely to undergo preconception genetic testing to avoid devastating disorders like Tay Sachs disease that are common in this population [28]. If the *Alu* insertion in *MAK* were added to Jewish disease panels the incidence of new cases of the disease could be reduced. However, such testing would also identify some completely presymptomatic affected adults near the beginning of their third decade of life who would have the greatest possible chance for a good outcome from gene augmentation therapy.

In summary, in this manuscript, we report the development of a novel, clinical grade gene augmentation vector for the treatment of *MAK*-associated RP and used patient-iPSC-derived retinal cells, a zebrafish morphant model, and normal Sprague Dawley rats to demonstrate safety and efficacy in vitro and vivo. Importantly, the pipeline used here is readily adaptable to many other heritable retinal degenerations and should allow us and others to accelerate the development of treatments for these conditions.

## Supplementary information

Supplemental Table 1

Supplemental Table 2

Supplemental Table 3

Supplemental Figure 1

Supplemental Figure 2
