# Supplementary material for: Development and biological characterization of a clinical gene transfer vector for the treatment of MAK-associated retinitis pigmentosa
Source: Gene Ther. 2021 Sep 14;29(5):259–88. doi: 10.1038/s41434-021-00291-5 (PMC9159943; doi:10.1038/s41434-021-00291-5)
Supplement: Supplementary file 1 — Supplemental Table 1 [file 41434_2021_291_MOESM1_ESM.docx]

| Supplemental Table 1. List of tissues that were weighed, subjected to gross evaluation and photographed, stored for qPCR based biodistribution, collected and preserved for histopathology, and subjected to microscopic evaluation for each animal at 1-month, and 3-months following vehicle or test article injection. | | | | | |
| --- | --- | --- | --- | --- | --- |
| Tissue | Organ Weights | Gross photo* | Stored for qPCR | Collected and Preserved | Microscopic Examination |
| Brain | X | X |  | X | X |
| Nose |  |  |  | X | X |
| Ethmoid sinus |  |  |  | X | X |
| Nasal lymphoid tissue |  |  |  | X | X |
| Tongue |  |  |  | X | X |
| Salivary gland |  |  |  | X | X |
| Incisors |  |  |  | X | X |
| Skeletal muscle (head) |  |  |  | X | X |
| Haired skin (head) |  |  |  | X | X |
| Peripheral nerve (head) |  |  |  | X | X |
| Eyes (with optic nerves) |  |  |  | X | X |
| Harderian gland |  |  |  | X | X |
| Olfactory bulb |  |  |  | X | X |
| Bone base of skull |  |  |  | X | X |
| Bone marrow (skull) |  |  |  | X | X |
| Pituitary gland |  | X |  | X | X |
| Lungs | X | X | X | X | X |
| Bronchus-associated lymphoid tissue |  |  |  | X | X |
| Spleen | X | X | X | X | X |
| Liver | X | X | X | X | X |
| Abdominal skin |  | X | X | X | X |
| Mammary glands |  | X | X | X | X |
| Stomach |  | X |  | X | X |
| Pancreas | X | X | X | X | X |
| Lymph node (abdominal) |  |  |  | X | X |
| Testis |  | X | X | X | X |
| Epididymis |  | X | X | X | X |
| Anterior prostate |  | X | X | X | X |
| Seminal vesicle |  | X | X | X | X |
| Urinary bladder |  | X |  | X | X |
| Duodenum |  | X | X | X | X |
| Jejunum |  | X |  | X | X |
| Ileum |  | X |  | X | X |
| Proximal Colon |  | X | X | X | X |
| Peyer’s patch |  | X |  | X | X |
| Distal colon |  | X |  | X | X |
| Leg |  | X | X | X | X |
| Sternum |  | X |  | X | X |
| Sciatic nerve |  |  |  | X | X |
| Spine - Cervical |  | X |  | X | X |
| Spine – Thoracic |  | X |  | X | X |
| Spine - Lumbar |  | X |  | X | X |
| Spine - Cauda Equina |  | X |  | X | X |
| Larynx | X | X |  | X | X |
| Trachea | X | X | X | X | X |
| Aorta | X | X |  | X | X |
| Esophagus | X | X |  | X | X |
| Ureter |  | X |  | X | X |
| Adrenal gland |  | X | X | X | X |
| Heart | X | X | X | X | X |
| Brown adipose tissue |  | X |  | X | X |
| White adipose tissue |  | X |  | X | X |
| Kidney | X | X | X | X | X |
| Thymus | X | X | X | X | X |
