# Supplementary material for: Development and biological characterization of a clinical gene transfer vector for the treatment of MAK-associated retinitis pigmentosa
Source: Gene Ther. 2021 Sep 14;29(5):259–88. doi: 10.1038/s41434-021-00291-5 (PMC9159943; doi:10.1038/s41434-021-00291-5)
Supplement: Supplementary file 2 — Supplemental Table 2 [file 41434_2021_291_MOESM2_ESM.pdf]

Supplemental Table 2: Clinical chemistry and hematology tests performed

Sample Type	Groups	Intervals	Parameters Evaluated
**Clinical Chemistry**	1-month<br>3-months	At time of sacrifice	• Total Protein (g/dl)<br>• Albumin (g/dl)<br>• Globulin (g/dl)<br>• Sodium (mEq/L)<br>• Potassium (mEq/L)<br>• Chloride (mEq/L)<br>• Total CO2 (mEq/L)<br>• Calcium (g/dl)<br>• Glucose (mg/dl)<br>• Alkaline Phosphatase (U/L)<br>• Alanine aminotransferase (U/L)<br>• Aspartate aminotransferase (U/L)<br>• Lactate dehydrogenase (U/L)<br>• Total bilirubin (mg/dl)<br>• Phosphorus (mg/dl)<br>• Blood urea nitrogen (mg/dl)<br>• Creatinine (mg/dl)<br>• Cholesterol (mg/dl)<br>• Triglycerides (mg/dl)<br>• Creatine Kinase (U/L)<br>• Uric Acid (mg/dl)<br>• HDL (mg/dl)
**Hematology**	1-month<br>3-months	At time of sacrifice	• RBC (M/uL)<br>• HGB (g/dL)<br>• HCT (%)<br>• MCV (fL)<br>• MCH (pg)<br>• MCHC (g/dL)<br>• RDW-CV (%)<br>• RET (K/uL)<br>• RET (%)<br>• PLT (K/uL)<br>• WBC (K/uL)<br>• NEUT (K/uL)<br>• NEUT (%)<br>• LYMPH (K/uL)<br>• LYMPH (%)<br>• MONO (K/uL)<br>• MONO (%)<br>• EO (K/uL)<br>• EO (%)<br>• BASO (K/uL)<br>• BASO (%)
