# Supplementary material for: Development and biological characterization of a clinical gene transfer vector for the treatment of MAK-associated retinitis pigmentosa
Source: Gene Ther. 2021 Sep 14;29(5):259–88. doi: 10.1038/s41434-021-00291-5 (PMC9159943; doi:10.1038/s41434-021-00291-5)
Supplement: Supplementary file 3 — Supplemental Table 3 [file 41434_2021_291_MOESM3_ESM.docx]

| Supplemental Table 3. QC and clinical release testing performed at each stage of the production pipeline depicted in Figure 5. | | |
| --- | --- | --- |
| QC 1 – Research grade plasmid testing | | |
| Test | | Analytical Method |
| Restriction Digest | | Gel electrophoresis |
| Plasmid Sequence | | Sanger sequencing |
| QC 2 – Research grade vector testing | | |
| Test | | Analytical Method |
| Physical Titer | | qPCR |
| Identity (capsid) | | Silver stain |
| QC 3 – Expression and toxicity analysis | | |
| Test | | Analytical Method |
| Expression level | | Western blot |
| Cellular cytotoxicity | | Microscopy |
| QC 4 – Efficacy and potency analysis | | |
| Test | | Analytical Method |
| Correction of disease phenotype in vitro | | Disease phenotype dependent (eg. cilia length defect – ICC and microscopy; ER-Stress – Western blot, ICC and ELISA; mitochondrial dysfunction – microscopy, oxidative phosphorylation, respiration, etc.) |
| Correction of disease phenotype in vivo | | Disease phenotype dependent (eg. cilia length rescue – ICC and microscopy; retinal function – ERG and OCT; preservation of retinal anatomy – ICC and microscopy of ONL thickness, etc.) |
| Local tolerance (wild type rats) | | Preservation of retinal anatomy – H&E and measurement of ONL thickness |
| QC 5 – Clinical plasmid release testing | | |
| Test | | Analytical Method |
| ABS 260/280 Ratio Purity | | UV Spectrophotometry |
| Concentration | | UV Spectrophotometry |
| Appearance | | Visual Inspection |
| DNA Homogeneity | | Densitometry via gel electrophoresis |
| Identity (size) | | Gel electrophoresis |
| Restriction Digest | | Gel electrophoresis |
| Endotoxin | | Kinetic Turbimetric LAL |
| Osmolality | | Osmometry |
| pH | | Potentiometry |
| Plasmid Sequence | | Sanger sequencing |
| Sterility Fungus/Bacteria and Bacteriostasis/Fungistasis | | Outgrowth Assay (USP<71>) |
| QC 6 - Clinical vector release testing | | |
| Test | Analytical Method | |
| Appearance | Visual Inspection | |
| Identity (vector) | Sanger sequencing | |
| Identity (capsid) | Silver stain | |
| Mycoplasma | qPCR | |
| Sterility: Fungus/Bacteria and Bacteriostasis/Fungistasis | Outgrowth assay (USP <71) – direct inoculation | |
| pH | Potentiometry | |
| Osmolality | Osmometry | |
| Physical Titer | qPCR | |
| Infectious Titer | *in vitro* transduction and qPCR | |
| Potency | *in vitro* transduction and Western Blot | |
| Residual Plasmid Titer | qPCR | |
| rcAAV | Cell transfection and qPCR | |
| Protein (total) | SDS-Page | |
| Protein (HEK) | ELISA | |
| Host Cell DNA | qPCR | |
| Residual Benzonase | ELISA | |
| Endotoxin | Endpoint Chromogenic LAL Assay | |
| Residual BSA | ELISA | |
| Empty Capsid Ratio | Transmission Electron Microscopy and analysis | |
| Adventitious Viral Agents Panel – Human, Simian, 9CFR, Bovine and Porcine viruses | Testing performed at contract research organization | |
