# Supplementary figures and images for: Development and biological characterization of a clinical gene transfer vector for the treatment of MAK-associated retinitis pigmentosa
Source: Gene Ther. 2021 Sep 14;29(5):259–88. doi: 10.1038/s41434-021-00291-5 (PMC9159943; doi:10.1038/s41434-021-00291-5)

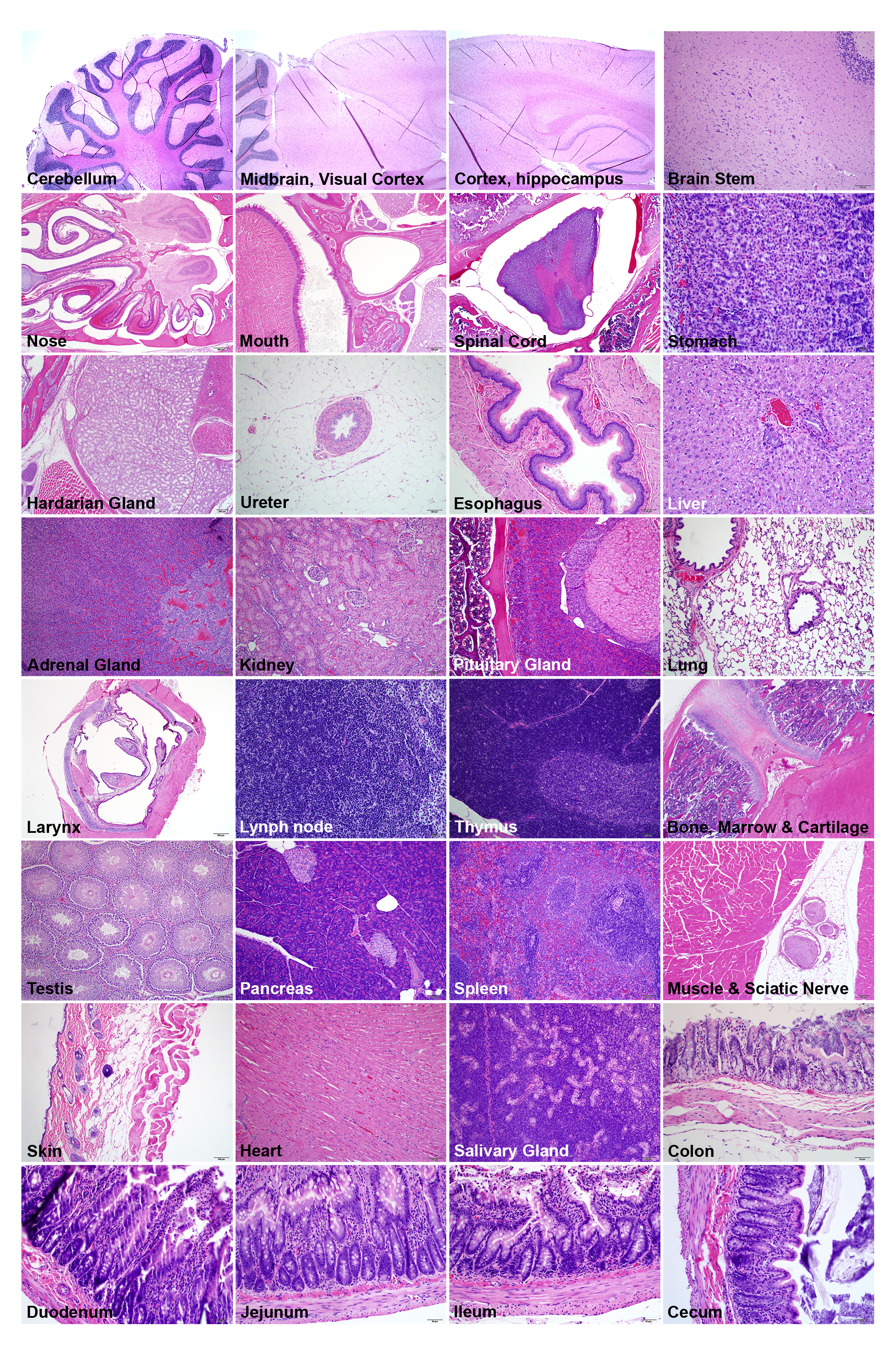

Supplement: Supplementary file 4 — Supplemental Figure 1 [file 41434_2021_291_MOESM4_ESM.jpg]

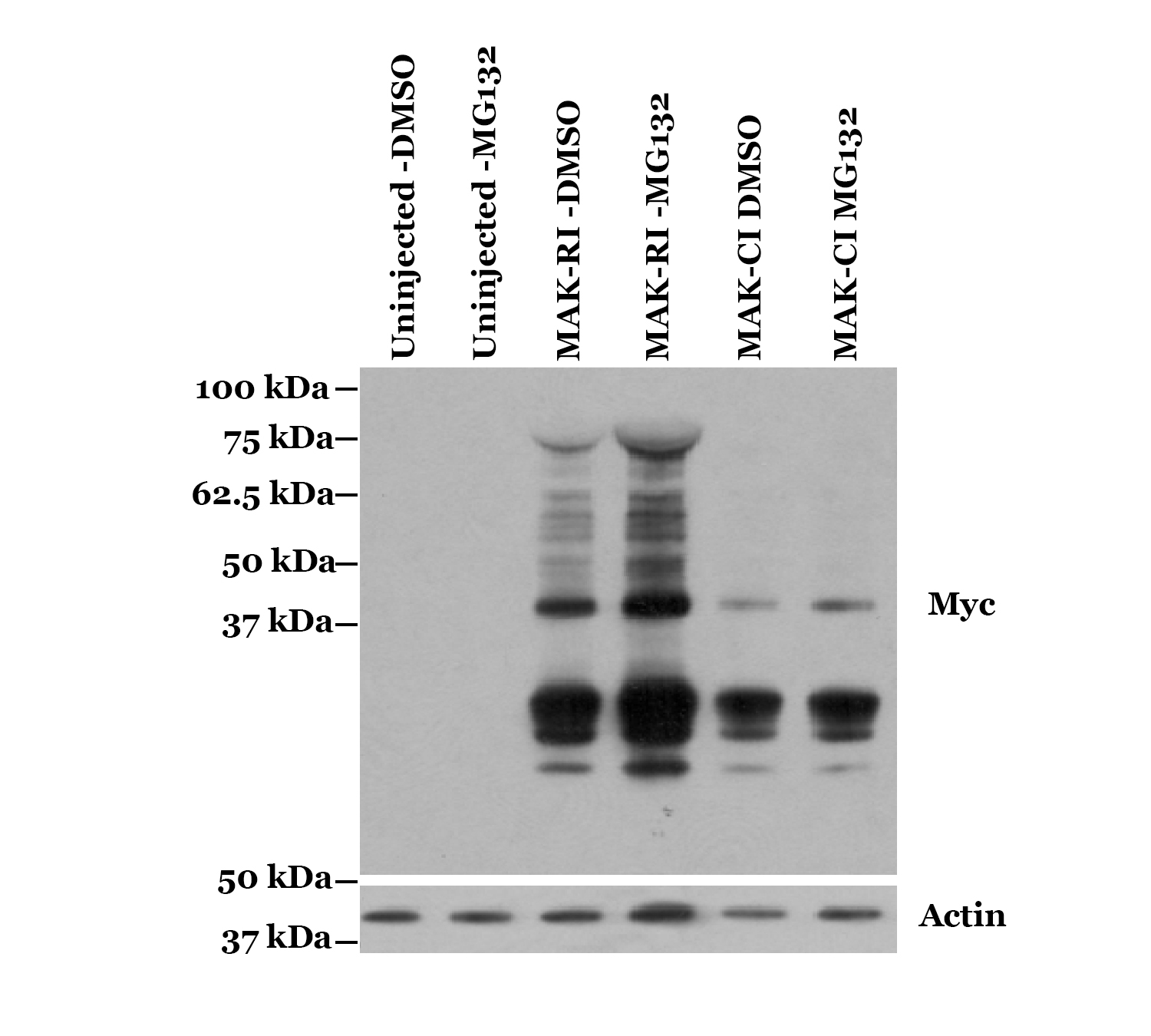

Supplement: Supplementary file 5 — Supplemental Figure 2 [file 41434_2021_291_MOESM5_ESM.jpg]
